# Enhancement of the mechanical properties in ultra-low weight SWCNT sandwiched PDMS composites using a novel stacked architecture

**DOI:** 10.1038/s41598-024-54631-7

**Published:** 2024-02-23

**Authors:** Pavithra Ananthasubramanian, Rahul Sahay, Nagarajan Raghavan

**Affiliations:** nano-Macro Reliability Laboratory (nMRL), Engineering Product Development (EPD) Pillar, Singapore University of Technology and Design, 8 Somapah Road, Singapore, 487372 Singapore

**Keywords:** Layered composites, Stack design, Carbon nanotube reinforced composites, Crack propagation, Nanoindentation analysis, Polymer nanocomposites, Soft materials, Polymers, Nanoscience and technology, Polymers, Rheology, Self-assembly, Composites, Mechanical properties, Characterization and analytical techniques, Design, synthesis and processing, Imaging techniques, Microscopy, Electronic devices, Sensors and biosensors, Materials science, Carbon nanotubes and fullerenes, Structural properties, Synthesis and processing, Chemistry, Materials chemistry

## Abstract

This study focuses on enhancing the mechanical properties of thin, soft, free-standing films via a layer-by-layer (LBL) fabrication process called LBL-FP. Soft polymer nanocomposite (PNC) thin films, combining polydimethylsiloxane (PDMS) and single-walled carbon nanotubes (SWCNT) at ultra-low loadings using a unique bottom-up LBL-FP, are examined. Two different structures of layered composites, (i) LBL PNCs- Layered composites with alternating layers of PDMS and SWCNT, (ii) Bulk PNCs- Layered composites with SWCNT dispersed in the bulk of PDMS, are comparatively investigated for their structural and mechanical properties. Silane-functionalized SWCNT strengthens the chemical bonding with PDMS, improving adhesion and dispersion. Mechanical analysis using nanoindentation, delamination, and dynamic analysis highlights the advantages of LBL PNCs with alternating layers of PDMS and SWCNT. Notably, LBL PNC (0.5 wt%) exhibits significant improvements, such as 2.6X increased nanoindentation resistance, 3X improved viscoelasticity, and (2–5)X enhanced tensile properties in comparison with neat PDMS. Due to this, LBL PNCs offer potential for soft, lightweight applications like wearables, electromagnetic interference shielding materials, and strain sensors while advancing composite thin film mechanics. The study emphasizes using a stacked architecture to produce PDMS-SWCNT multilayered PNCs with improved mechanics utilizing ultra-low concentrations of SWCNT. This first-of-its-kind stack design facilitates possibilities for lightweight composites utilizing less fillers. The LBL assembly involves the stacking of alternating layers of different materials, each contributing specific properties to enhance the overall strength and toughness of the structure.

## Introduction

Polydimethylsiloxane (PDMS) is one of the most widely explored elastomers in silicone polymers. It has been extensively adopted as a substrate material for manufacturing lab-on-a-chip and flexible electronic devices^1^. PDMS has been significantly studied in fabricating the substrate of wearable strain sensors which can be attached to the skin to detect human joints’ large-scale motion^2^. On the commercial front, Sylgrad 184 from Dow Corning is one of the most sorted versions of PDMS despite it being costlier than the other versions of silicone polymers. However, it offers several primary advantages over other flexible substrate materials. These materials include polyethylene naphtholate, polycarbonate, etc. from standard brands like Merck-Sigma Aldrich. The advantages of Sylgrad 184 include low cost, ease of material handling, fast simple fabrication, non-toxicity, chemical inertness, and optical transparency. The mechanical reliability of PDMS is crucial in all the applications of Sylgrad 184. Young’s modulus of Sylgrad 184 PDMS has been reported with significant variation from ~ 1 MPa^3,4^ to over 3 MPa^5^ at its unmodified conditions^6^.

The insertion of carbon nanostructures, notably carbon nanotubes (CNT), as a filler in the PDMS matrix has been extensively investigated to increase the strength and Young’s Modulus of PDMS at the site of application and during the operation of PDMS-based material systems as a device^7–27^. CNT-reinforced PDMS polymer nanocomposites have recently been reported to find extensive applications in strain sensors and actuator applications attributing to their improved mechanics^28,29^. In a recent report by Alam, M. N. et al.^30^ the synergetic reinforcement provided to PDMS by CNT (to improve the tensile strain) and MOS_2_ (to improve the fracture strain) is discussed. CNTs are one of the most sorted choices of reinforcing fillers for PDMS to achieve multifunctional property enhancement (mechanical, electrical, etc.). This can be attributed to its outstanding electrical, mechanical, physical, and electrochemical properties, such as high electrical conductivity (10^6^–10^7^ s/m), significant Young’s Modulus of 0.45 TPa (10^12^ Pa), and favorably high surface-to-volume ratio (up to 550 m^2^/ cm^3^)^23^. In PDMS-based composite systems, a high weight percentage loading of CNT in the range from 1 wt% to 7 wt% is usually investigated to achieve mechanical and electrical percolation^15,16,18,24,26,27^. Certain specific challenges prevail in the existing PDMS-CNT composite thin films:

i. Inhomogeneous dispersion and dispersion stability of the low-density CNT filler in the bulk of the viscous PDMS matrix.
ii. Crack propagation and failure of the thin film along the thickness (z-axis) of the film due to the lower stiffness of thin films along the z-axis.

These challenges limit the possibilities of realizing this composite system widely on the commercial front. During upscaling the process from a laboratory scale to an industrial scale, the dispersion of CNT in Sylgrad 184 PDMS is a challenge that limits the utilization of the material system^31–34^. Most of the mechanical tests conducted on PDMS-CNT-based composite thin films evaluate the tensile strength, elongation at break, and tensile modulus or compressive strength of the composites^2,6,8,9,19–24,26,35–40^. Though these results are reliable in terms of quantifying the 3D mechanical properties of the thin films, improving the mechanical robustness, especially stiffness, in the z-direction along the thickness of the film is crucial. These properties can be improved by simply altering the design of the composite thin film. We propose that the mechanical properties of the thin film in the z-direction can be amplified when the distribution of a mechanically reliable filler, SWCNT, along the thickness of the composite thin film is controlled.

To address this issue, the fabrication of composite thin films using a layer-by-layer fabrication process (LBL-FP) is proposed. In this design, the assembly of alternating layers of PDMS and SWCNT through a simple solution-based bottom-up approach to fabricate composite thin films is attempted. In this method of fabrication, SWCNT and PDMS are individually stacked as alternating layers over each other. Therefore, PDMS and SWCNT need not be mixed together which overcomes the challenge of dispersing the low dense SWCNT homogeneously in a viscous PDMS matrix. However, it is important to note that alternating layers of PDMS and SWCNT in the composite thin film will result in non-isotropic mechanical properties. This approach is specifically proposed to improve the mechanical robustness, especially stiffness, in the z-direction without compromising the fundamental mechanical properties along the x–y plane.

LBL fabrication of soft films and multi-layered polymer nanocomposite (PNC) architectures is a field that is widely explored to understand the interfacial properties and structural mechanics of composite assemblies^41–45^. LBL fabrication of PDMS-based polymer nanocomposite devices have been of specific interest to researchers in the last decade^8,46–55^. Most of the reports discuss the LBL assembly and sandwich structures of PDMS-based composite systems in the context of gas separation^48^, strain sensors^8,51^, microfluidic devices ^46,55^ solvent pervaporation^49^, wearable biosensors^50^, and flexible energy storage devices^11,54^. This is one of the first reports to extensively discuss the mechanics of PDMS-SWCNT composite thin films fabricated through a facile and novel LBL- FP.

This work investigates comparatively the mechanical properties in LBL PNCs with bulk PNCs. This work is also one of the first reports that evaluates the mechanics of silylated single wall CNT-reinforced PDMS composites. Silane functionalized single-walled carbon nanotubes (Sily-SWCNT) have helped in achieving better dispersion and chemical bonding between SWCNT and PDMS^56^. Attributed to the presence of rigid and high-modulus Sily- SWCNT in combination with the strong interlayers' adhesion from the LBL curing, the LBL assembled composite thin films exhibit an enhancement in the viscoelastic behavior with improved mechanical reliability. In LBL stacked thin film composites, there is a noticeable improvement in the distribution of fillers across the thickness of the composite, along the z-plane. This improvement is evident in the mechanical properties of the films investigated along the z-plane (nanoindentation and delamination analysis) and in all three axes (dynamic mechanical analysis). FESEM imaging and FTIR analyses are conducted to confirm the LBL structure and covalent chemical bonding in the material system. Nanoindentation analyses, delamination analyses, and dynamic mechanical analyses are conducted on composite thin film samples to quantify the mechanical properties.

## Materials and methods

### Materials

PDMS (Sylgrad 184) was purchased from Dow Corning, USA. Carboxylic acid functionalized single-walled carbon nanotubes (COOH- SWCNT) (> 90% carbon basis, D × L 4–5 nm × 0.5–1.5 μm, bundle dimensions), and 3-Aminopropyltriethoxy silane, 99% (APTES) were purchased from Merck- Sigma Aldrich, Singapore.

### Methods

#### Silylation of carboxylic acid functionalized single-walled carbon nanotubes

Silylation in SWCNT is proposed as a nanometric coupling agent for SWCNT with polymer matrices. This procedure is an initiative to develop new nano arrangements and improve adhesion between SWCNT and PDMS. The silylation modification is performed on the COOH-SWCNT to convert its hydrophilic to hydrophobic surface chemistry, promoting the dispersion, and interactions between the Sily-SWCNT and hydrophobic PDMS matrix in the composite layers. The effect of this chemical bonding is realized in the improved mechanical properties of the composite structures even at ultra-low weight percent loadings (0.05–1 wt%) of SWCNT in the matrix due to the covalent chemical bonding between the Sily-SWCNT fillers and PDMS matrix. In addition, layer-by-layer fabricated thin film composites improve the effect of SWCNT reinforcement in the matrix even at low weight percentage loadings of SWCNT in the matrix (to be discussed in detail in the subsequent sections). The silylation of SWCNT reaction was conducted based on a previously reported procedure^57,58^.

#### Dispersion of Sily-SWCNT in methanol

We present a comparative study of the mechanics of Sily-SWCNT reinforced PDMS composite thin films between LBL PNCs and bulk PNCs. Both types of composites were fabricated with four weight percentage ultra-low loadings of Sily-SWCNT on PDMS- (i) 0.05 wt%, (ii) 0.2 wt%, (iii) 0.5 wt% and (iv) 1 wt%. For both types of composites, Sily-SWCNT were first dispersed in methanol attributed to its high polarity (0.762) and low boiling point (64.96 °C)^59,60^. Sily-SWCNT dispersed in methanol were then dispersed in PDMS to achieve a uniform distribution of SWCNT in the PDMS matrix. Dispersion of Sily-SWCNT in methanol was realized using a facile ultrasonication procedure at 25 Hz for 30 min using Kunshan ultrasonic instrument (KQ3200DA) and the bath temperature was maintained at 25 °C.

#### Layer-by-layer fabrication process (LBL-FP) of alternate layers of PDMS-SWCNT composite thin films (LBL PNCs)

The measured weight of Sylgrad 184 PDMS elastomer base (Sylgrad A) and Sylgrad 184 curing agent (Sylgrad B) were mixed in the standard weight ratio of 10:1. The base elastomer and the curing agent were manually mixed using a glass stick. The mixture was degassed in vacuum for 10 min to eliminate air bubbles. Measured volumes of Sily-SWCNT dispersion in methanol in required concentrations were kept ready. The dispersion was subjected to ultrasonication in an ultrasonic cleaner (2000 mL, EQ-VGT-1620QTD) at 25 Hz and the bath temperature was maintained at 25 °C throughout the LBL-FP of composite thin films. Table 1 depicts the parameters used for the fabrication of samples using an LBL- FP inside a Class-1000 clean room.

**Table 1:** Experimental design for the layer-by-layer fabrication of PNCs.

S. no	Sample code	Sample description	PDMS weight (g)	Sily-SWCNT weight (%)	No. of PDMS layers	Weight of PDMS/ layer (g)	No. of Sily-SWCNT layers	Weight of Sily-SWCNT/ layer (mg)	Concentration of Sily-SWCNT dispersion	Volume of Sily-SWCNT dispersion/ layer (mL)
1	Neat PDMS	7 layers of neat PDMS	7	0	7	1	0	0	NA	NA
2	LBL PNC (0.05 wt%)	7 alternating layers of PDMS and SWCNT composite with 0.05 wt% SWCNT loading	7	0.05	7	1	7	0.5	1 mg/mL	0.5
3	LBL PNC (0.2 wt%)	7 alternating layers of PDMS and SWCNT composite with 0.2 wt% SWCNT loading	7	0.2		1		2	3 mg/ mL	0.7
4	LBL PNC (0.5 wt%)	7 alternating layers of PDMS and SWCNT composite with 0.5 wt% SWCNT loading	7	0.5		1		5	5 mg/ mL	1
5	LBL PNC (1 wt%)	7 alternating layers of PDMS and SWCNT composite with 1 wt% SWCNT loading	7	1.0		1		10	10 mg/ mL	1

**Figure 1:**
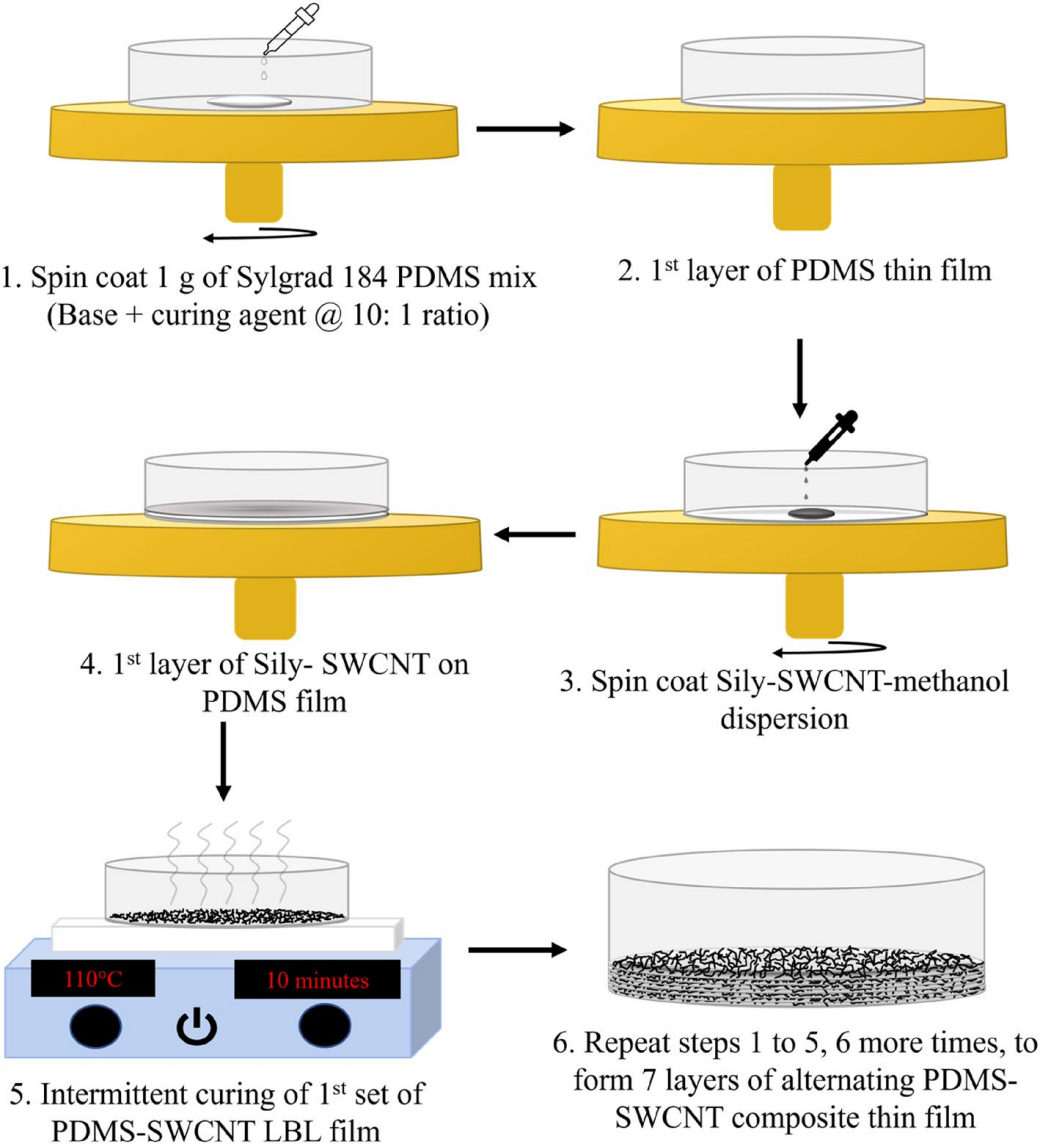
Schematic of the fabrication process of LBL PNCs.

The fabrication of LBL PNCs employs a facile spin coating technique using a Laurell WS-650Mz-23NPPB spin coater. All samples were fabricated as 7 stacked alternating layers of PDMS and Sily-SWCNT. Each PDMS layer was fabricated by spin coating 1 g of PDMS elastomer mix to form a ~ 25 µm thick film. The required volume of SWCNT dispersion is consecutively spin-coated on the PDMS layer. Each PDMS-SWCNT layer was intermittently cured on a hot plate (hot plates with magnetic stirring, EQ-SH-3) at 110 °C for 10 min. As proposed by T. Xia et al.^51^ the surface silylation of SWCNT improves the dispersion of SWCNT in the hydrophobic PDMS matrix. The proposed layer-by-layer curing approach gives critical interfacial adhesion to the inter-laminates, which enables the adequate integration of the inter-layers for the multi-layered composites. Following this method, every PDMS-SWCNT layer was successively spin-coated and intermittently cured before the casting of the of the next layer. Figure 1 schematically depicts the LBL-FP of the PNCs discussed in Table 1.

The LBL-FP of neat PDMS followed the same process after skipping the steps of assembly of SWCNT in every alternate layer. After manufacture, all samples underwent four hours of curing at 70 °C in a vacuum oven to guarantee that the PDMS had fully cured, and that all remaining methanol had been removed from the samples.

#### Layer-by-layer fabrication process (LBL-FP) of *Bulk* PDMS-SWCNT thin film composites (Bulk PNCs)

A measured weight of Sylgrad 184 PDMS elastomer base (Sylgrad A) was taken in a glass beaker. The required volume of Sily-SWCNT dispersed methanol to achieve the required weight percent loading of SWCNT on PDMS was added to the PDMS base. This mixture was stirred at 300 rpm at 70 °C to evaporate methanol from the mixture using a magnetic stirrer (Hot plates with magnetic stirring, EQ-SH-3).

**Figure 2:**
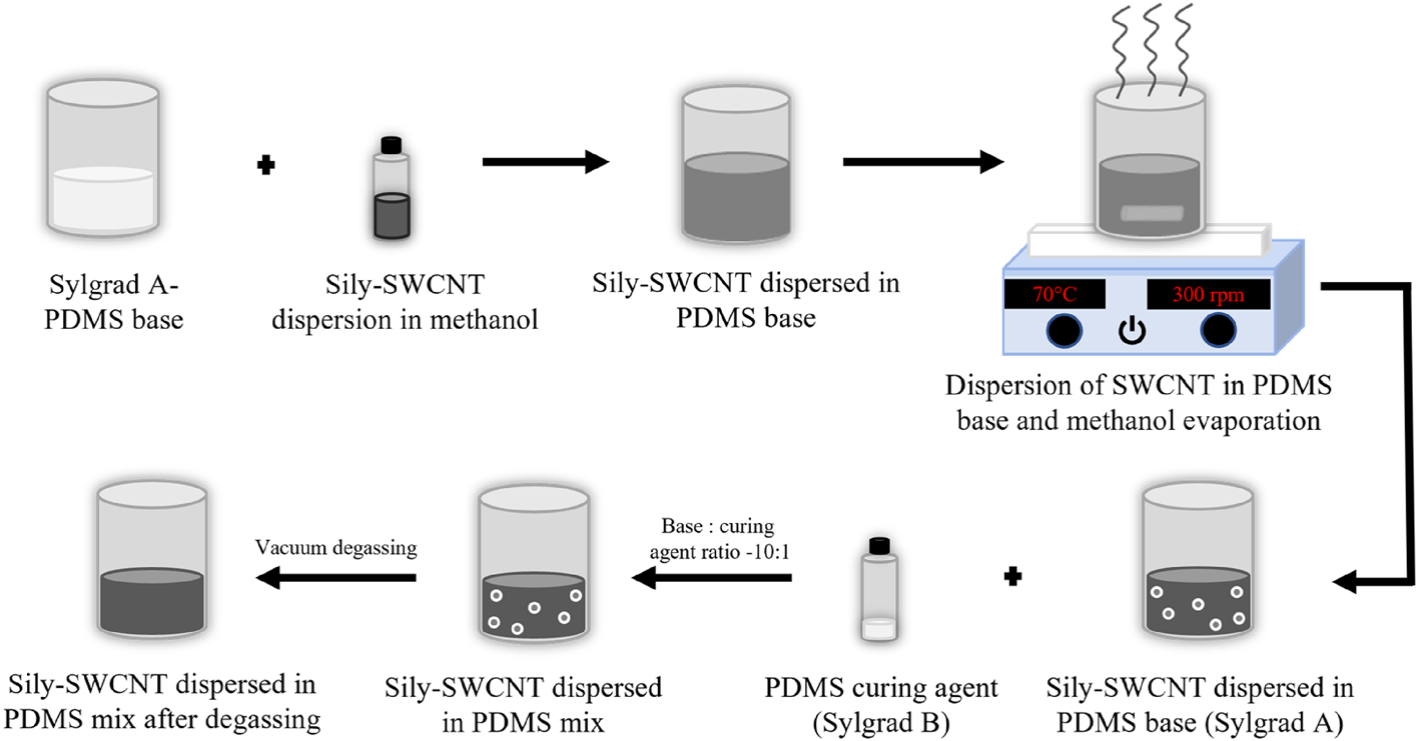
Schematic representation of the dispersion process of Sily-SWCNT in PDMS to fabricate bulk PNCs.

The curing agent for Sylgrad 184 PDMS, Sylgrad B, was measured and added to the SWCNT dispersed PDMS base. The base: curing agent ratio was 10:1. The mixture was mixed well to ensure the homogeneous distribution of the curing agent in the PDMS base. The mixture was degassed in vacuum for 10 min to remove air bubbles. The schematic of this procedure is represented in Fig. 2.

After the dispersion of SWCNT in bulk PDMS mix in the required weight percentages, bulk PNCs were fabricated LBL, along with a LBL curing approach as discussed in section “Layer-by-layer fabrication process (LBL-FP) of alternate layers of PDMS-SWCNT composite thin films (LBL PNCs)”, using a facile spin coating technique. The experimental design involved in the fabrication of bulk PNCs is shown in Table 2.

**Table 2:** Experimental design for the LBL-FP of Bulk PNCs.

S.No	Sample code	Sample description	PDMS weight (g)	Sily-SWCNT weight %	Sily-SWCNT weight (mg)
1	Bulk PNC-0.05	7 layers of PDMS-SWCNT composite with 0.05 wt% SWCNT loading	7	0.05	3.5
2	Bulk PNC-0.2	7 layers of PDMS-SWCNT composite with 0.2 wt% SWCNT loading	7	0.2	14
3	Bulk PNC-0.5	7 layers of PDMS-SWCNT composite with 0.5 wt% SWCNT loading	7	0.5	35
4	Bulk PNC-1.0	7 layers of PDMS-SWCNT composite with 1 wt% SWCNT loading	7	1.0	70

All four composite samples were fabricated as 7 stacked layers of bulk PDMS-SWCNT composite thin films. Each layer was fabricated by spin coating 1 g of the mix at 1000 rpm for 5 min to form a ~ 25 µm thick film. Each layer was also intermittently cured at 110 °C for 10 min. Following this method, each layer was successively spin-coated and intermittently cured before the casting of the next layer.

**Figure 3:**
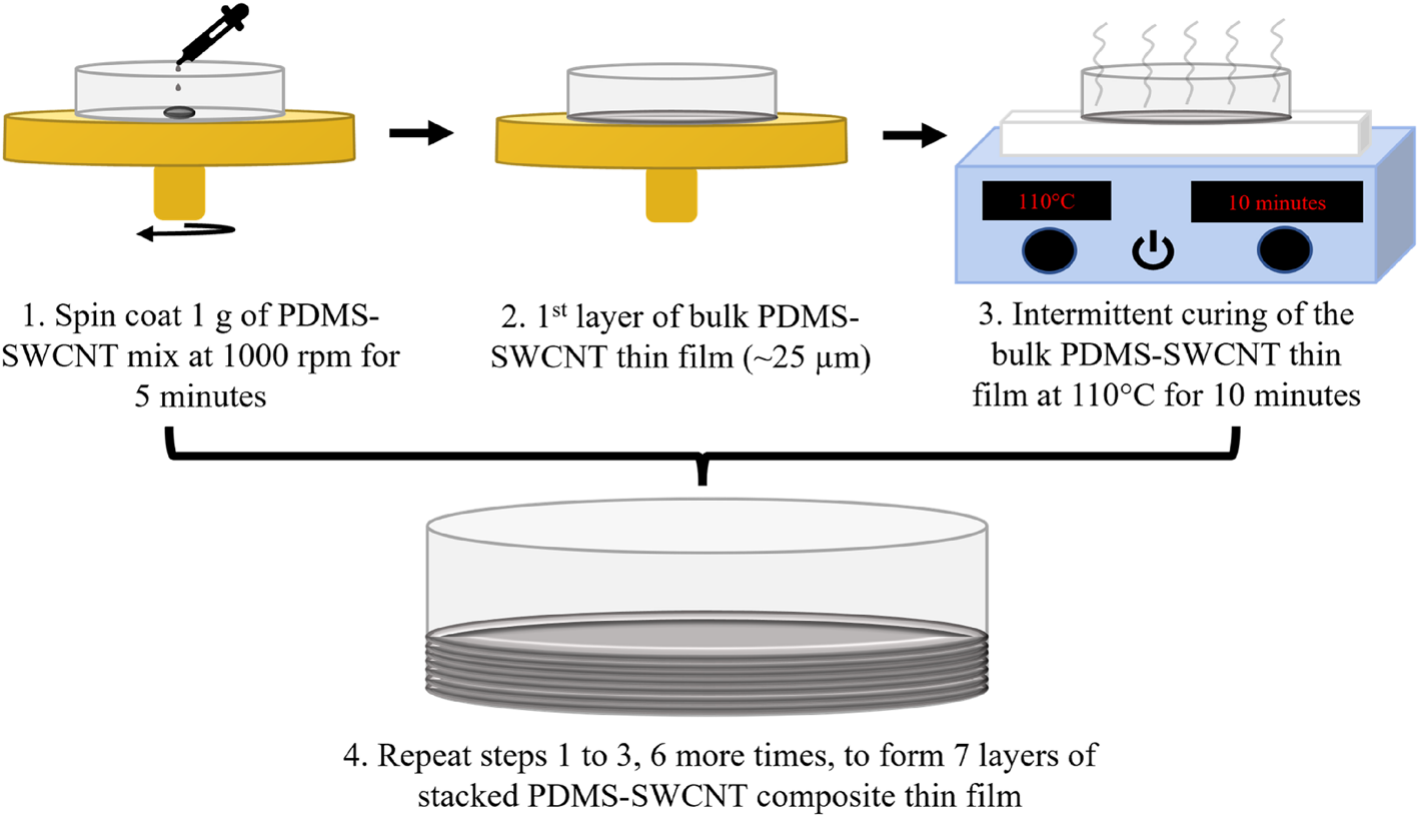
Schematic of the fabrication process of bulk PNCs.

The schematic shown in Fig. 3 is a representation of the LBL-FP of bulk PNCs, which shall act as specific controls against their respective LBL PNCs. All samples were cured inside a vacuum oven at 70 °C for 4 h to ensure complete curing of PDMS in the composites.

**Figure 4:**
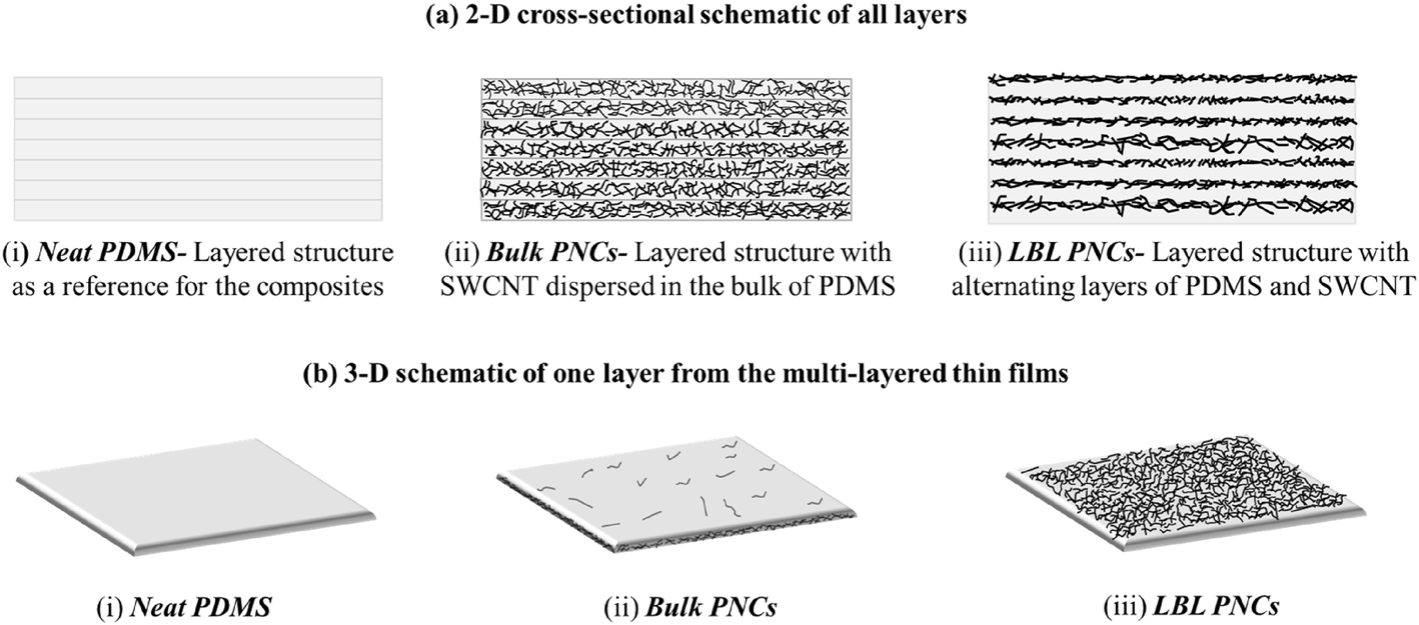
(**a**) 2-D schematic of all the multi-layered thin films fabricated through a novel LBL- FP and (**b**) 3-D schematic of one layer from the multi-layered thin films.

A 2-D schematic of the multi-layered neat PDMS, LBL PNCs and Bulk PNCs are shown in Fig. 4a. A 3-D schematic of the top layer from multi-layered thin films is also represented in Fig. 4b.

As indicated in Fig. 4a, it is understood that in bulk PNCs, the multi-layered thin films have SWCNT dispersed in the bulk of PDMS in every layer of the thin film. However, in LBL PNCs, PDMS and SWCNT are alternatively stacked as a multi-layered thin film. Figure 4b is a schematic illustration of how a given wt% of SWCNT loading on PDMS gets distributed differently in a 3-D dispersion (bulk PNCs) and 2-D dispersion (LBL PNCs). In LBL PNCs, as SWCNT is spin coated on the top surface of every PDMS layer, the distribution of SWCNT is majorly on the surface.

**Figure 5:**
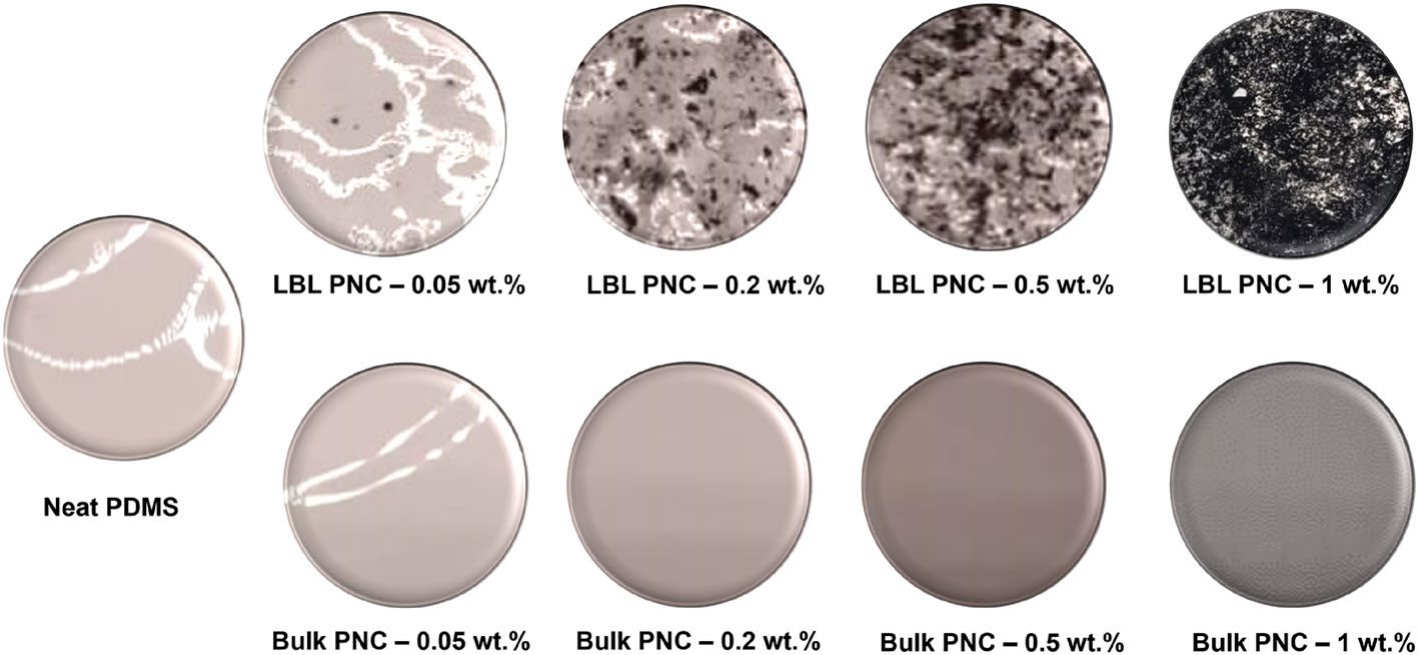
Top-view photographic image of the fabricated polymer nanocomposite samples. The figure shows variation in the distribution of SWCNT on the top surface of the samples. LBL PNCs have SWCNT on the top surface while Bulk PNCs have SWCNT dispersed in the bulk of PDMS leading to the difference in the contrast of the images.

A top-view photograph of all the nine samples that were jointly discussed in Tables 1 and 2 are shown in Fig. 5. The photograph reveals the variation in distribution of Sily-SWCNT on the top surface of the samples.

#### Field emission scanning electron microscopy (FESEM)

FESEM analysis was conducted using JEOL JSM-7600F. Firstly, the polymeric samples were freeze-dried at -100 °C with 40 mT pressure for 24 h in a freeze dryer (VirTis Bench Top Pro with Omnitronics™). The freeze-dried samples were manually broken using forceps. The samples were pasted on a cross-sectional sample stub and were sputtered with gold. The gold-sputtered samples were then investigated under FESEM to understand the LBL morphology and micro-structural properties along the cross-section of each sample.

#### Fourier transform infrared spectroscopy (FTIR)

Thermo Scientific Nicolet iS20 was used to scan the samples at room temperature from 400 cm^-1^ to 4000 cm^-1^. FTIR analysis was comparatively conducted on the untreated COOH-SWCNT and Sily-SWCNT powders. Attenuated Total Reflectance- Infrared Spectroscopy (ATR-IR) analysis was conducted on polymeric thin films.

#### Nanoindentation analysis

Nanoindentation studies were conducted using Hysitron TriboIndenter TI 950. This is a unique tool that is used extensively to conduct localized analysis of mechanical properties of composite thin films on a nano-micro scale. Nanoindentation of PDMS based thin films is a common technique used to evaluate its viscoelastic properties^61,62^. All the samples were indented under a constant displacement control mode using a Berkovich tip to generate loading–unloading data in the form of force–displacement curves. This information is used to estimate the plastic work (*W*_*p*_), reduced modulus (*E*_*r*_) Young’s Modulus (*E*), hardness (*H*) and contact stiffness (*S*_*c*_). All the samples were indented to a depth of 1000 nm. The loading rate was 5 nm/s, and the unloading rate was 10 nm/s. Each test was conducted for a minimum of 50 points for each sample to ensure repeatability. To prevent the Mullins effect, a fresh location on the sample was chosen for each individual nanoindentation test^61,63,64^. The reduced elastic modulus (*E*_*r*_), Young’s Modulus (*E*), hardness (*H*) and contact stiffness (*S*_*c*_) of the thin film samples are evaluated from the slope of the unloading curves using previously reported standard analysis techniques^61,65–69^. The analysis methods and the equations used for the calculations are discussed in detail in section S2 of supplementary information.

#### Delamination analysis

The delamination analysis was conducted using a DTS delaminator-Adhesion testing system to estimate the inter-layer bonding strength (B.S.) and time taken for delamination. The delaminator adhesion test system is a high-precision micro-mechanical test system with full computer control and data analysis used for thin-film adhesive and cohesive fracture energy testing. The testing parameters included a pre-load of 0.001 N and the force ramp was set at 0.01 N/ min. The test was conducted at room temperature on all samples. Each thin film was tested using triplicate samples in the DTS delaminator unit and the average values were used to analyse the results. The length and width of the samples were (10 × 10) mm for all samples.

#### Dynamic mechanical analysis

Dynamic mechanical analysis was conducted using a TAQ800 dynamic mechanical analyzer (DMA). All the tests were conducted at 25 °C. The dynamic mechanical test was conducted to estimate the bulk stiffness (*k*) and storage modulus (*E’*) values of the samples. The samples were tested under a multi-strain mode with a strain sweep of 0.1 to 1000 µm at a frequency of 20 Hz. The samples were also subjected to a preload force of 0.001 N. The length and width of the samples were (15 × 3) mm for all samples.

## Results and discussion

### Surface morphological analysis using field emission scanning electron microscopy (FESEM)

**Figure 6:**
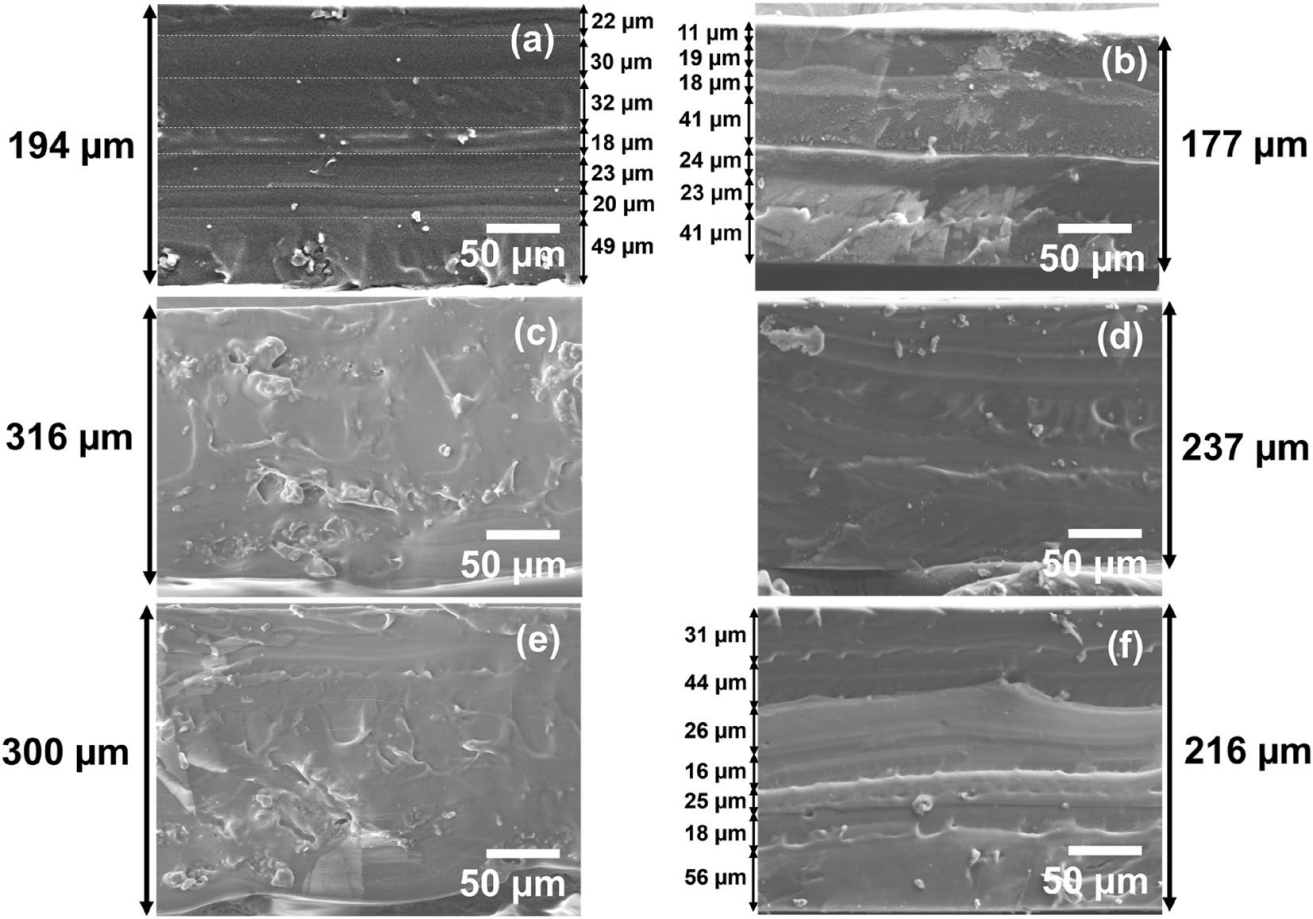
Cross-sectional FESEM imaging of the LBL and bulk PNC samples: (**a**) LBL PNC (0.2 wt%), (**b**) Bulk PNC (0.2 wt%), (**c**) LBL PNC (0.5 wt%), (**d**) Bulk PNC (0.5 wt%), (**e**) LBL PNC (1 wt %), and (**f**) Bulk PNC (1 wt%). The figures reveal the multilayered surface morphology from a cross-sectional view.

Figure 6a–f presents cross-sectional FESEM images of LBL and bulk PNC samples with different weight percentages, ranging from 0.2 wt% to 1 wt%. From the FESEM images, it is observed that the layers in the LBL PNCs are not as obviously visible as they are in the bulk PNCs, which may be attributed to the improved intermixing of PDMS layers during the fabrication which has resulted in improved mechanical properties of LBL compared to bulk and neat samples. In the LBL PNCs, alternating layers of PDMS and SWCNT were spin-coated separately. SWCNT dispersed in methanol is used to form the SWCNT layer on top of spin-coated PDMS layer. Each PDMS-SWCNT layer is also subjected to layer-by-layer curing as discussed in section “Layer-by-layer fabrication process (LBL-FP) of alternate layers of PDMS-SWCNT composite thin films (LBL PNCs)”. Due to the evaporation of methanol during the LBL curing of PDMS-SWCNT in each layer, the uniformity in the height profile of each layer gets distorted. This increases the roughness of each PDMS-SWCNT layer in the microstructure. Due to the increased roughness of each layer created during the methanol evaporation step, the interface bonding of the consequent PDMS layer with the previous PDMS-SWCNT is superior to the interface bonding of the layers in the bulk PNCs. However, in the bulk PNCs, each layer is clearly seen through FESEM. This is due to the absence of the methanol evaporation process during the intermittent curing of each layer.

Furthermore, each layer's thickness is not shown to be the same, either in LBL PNCs or bulk PNCs. This could be explained by the processing conditions that the composite thin films are put through. For instance, compared to the other layers in the LBL PNC, the first layer (the bottom layer) is subjected to more cycles of spinning and intermittent curing. The non-equivalent thickness of each layer in the composite thin films (LBL and bulk) is a result of these manufacturing conditions. It can also be noted from the layer thickness measurements in Fig. 6a,b,f that the average layer thickness of each layer in the samples is between 25 and 30 µm which is comparable to the expected thickness of 25 µm as per the process settings used to fabricate the thin films.

**Figure 7:**
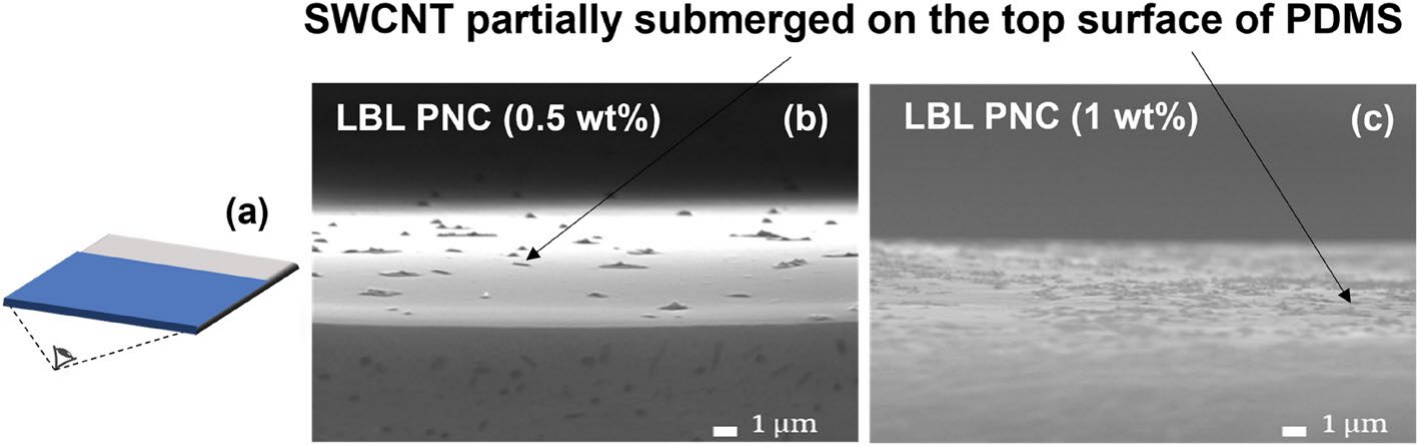
Top-surface image from a cross-sectional angle: (**a**) viewing angle of the top surface of thin films from a cross-sectional angle (**b**) LBL PNC (0.5 wt%), (**c**) LBL PNC (1 wt%). The figures reveal the CNT network density on the top surface of the thin films.

The top surface of the LBL PNCs from a cross-sectional angle is compared using FESEM (Fig. 7b,c) for the SWCNT wt% of 0.5, and 1 respectively. Figure 7a is a schematic of the viewing angle of the top surface of the LBL PNCs from a cross-sectional view. From Fig. 7b and c, it is observed that, as anticipated, the SWCNT density on the top surface of the LBL PNCs increases with increasing weight percentage loadings in the composite. The microstructure reveals a partial submergence of the nanotubes over each layer of the PDMS matrix, representing a ‘*needle-in-a-haystack*’ type of reinforcement of the nanotube in the matrix. The top surface analysis of the samples is also conducted, and the images are discussed in the supplementary information in Fig. S1.

### Analysis of chemical functional groups on the samples using Fourier transform infrared spectroscopy (FTIR)

**Figure 8:**
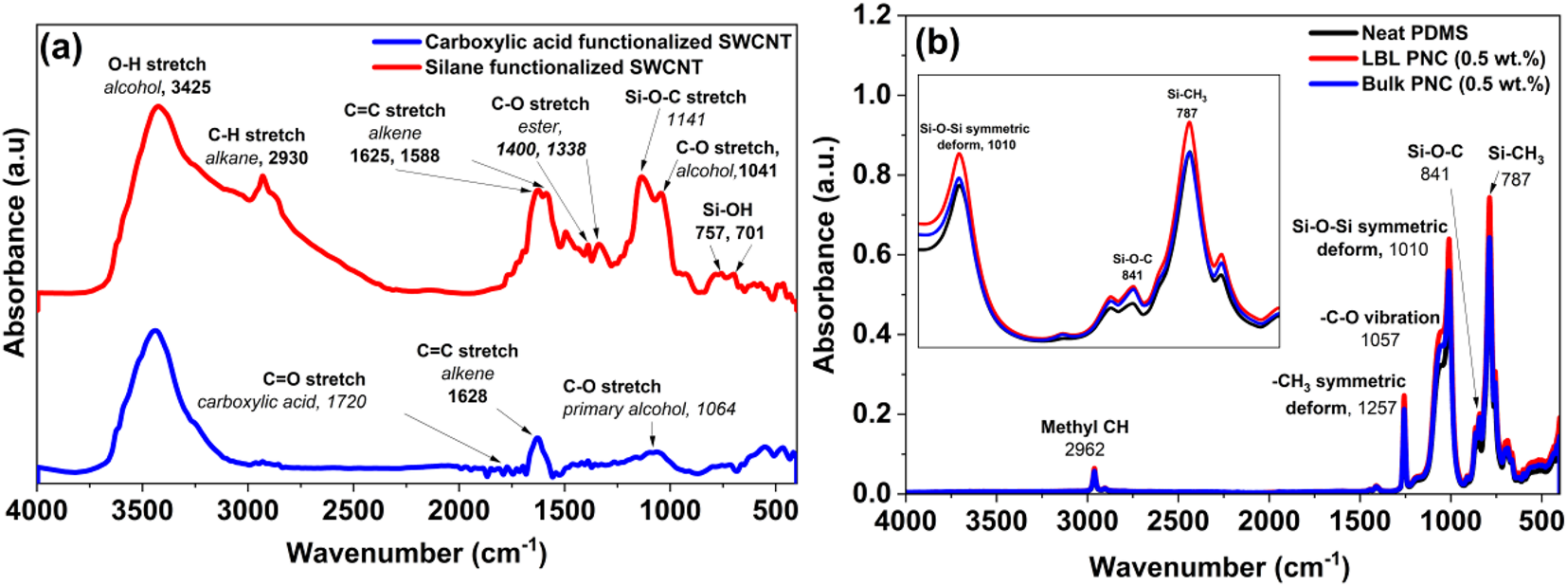
(**a**) Comparative FTIR analysis of COOH-functionalized SWCNT and Silane functionalized SWCNT (**b**) Comparative ATR-IR analysis of neat PDMS, Silane functionalized SWCNT reinforced LBL PNC (0.5 wt%) and bulk PNC (0.5 wt%). The FTIR plots reveal the effective silane functionalization of SWCNT (Fig. 8(**a**)) and the chemical adhesion between Sily-SWCNT and PDMS (Fig. 8(**b**)).

Figure 8a shows the comparative FTIR analysis of COOH-SWCNT and Sily-SWCNT. As previously reported, in the –COOH functionalized SWCNT, the vibrations at 1729 cm^−1^ and 1065 cm^−1^ are attributed to the C=O and C–O stretches from the –COOH functionalization group. The characteristic absorption peaks at 3425 cm^−1^ are from the hydroxyl group (–OH) in the carboxylic group^57^. In the FTIR plot of Sily-SWCNT, the peak at 1141.2 cm^−1^ is from Si–O–C vibration confirming the formation of a covalent chemical bond between silane and SWCNT. The weak signals from 757 and 701 cm^−1^ indicate the Si–OH bond vibrations. It is also interesting to note that the weak signal at 1338 cm^−1^ is due to O–H bond deformation bend in –COOH functional group. This is because the silane groups get attached to the –OH functional groups on the surface of the COOH functionalized SWCNT^57,70^.

Figure 8b is a comparative representation of the ATR-IR analysis of neat PDMS, LBL PNC (0.5 wt%), and bulk PNC (0.5 wt%). The peaks in neat PDMS and the composites are comparatively analyzed and marked in the plot. The peaks at 2962 cm^−1^, 1257 cm^−1^, and 1057 cm^−1^ are from the vibrations of methyl CH groups, -CH_3_ asymmetric deformation, and –C–O stretching respectively, corresponding to the groups in PDMS^71^.

The peaks at 1010 cm^−1^, 841 cm^−1^, and 787 cm^-1^ refer to Si–O–Si symmetric deformation, Si–O–C vibration, and Si-CH_3_ vibrations respectively^71^. In the inset in Fig. 8b, it is important to note that the intensity of the Si–O–Si, Si–O–C, and Si–CH_3_ bonds have increased for both the composites with respect to neat PDMS. This is due to the presence of silane groups in silanized SWCNT in the composite. The increase in the intensity of the Si–O–Si bond and Si–CH_3_ bond confirms the chemical bonding between silanized SWCNT and PDMS matrix^72^.

### Nanoindentation analysis

#### Nanoindentation analysis and mechanics of viscoelastic behavior

Nanoindentation analysis has been conducted on all the thin film samples using a Berkovich tip. The details about the Berkovich tip and a schematic of the interaction of the Berkovich tip with the top surface of the three types of thin films in this study: (i) Neat PDMS, (ii) Bulk PNCs and (iii) LBL PNCs are discussed in section S2 of the supplementary information.

**Figure 9:**
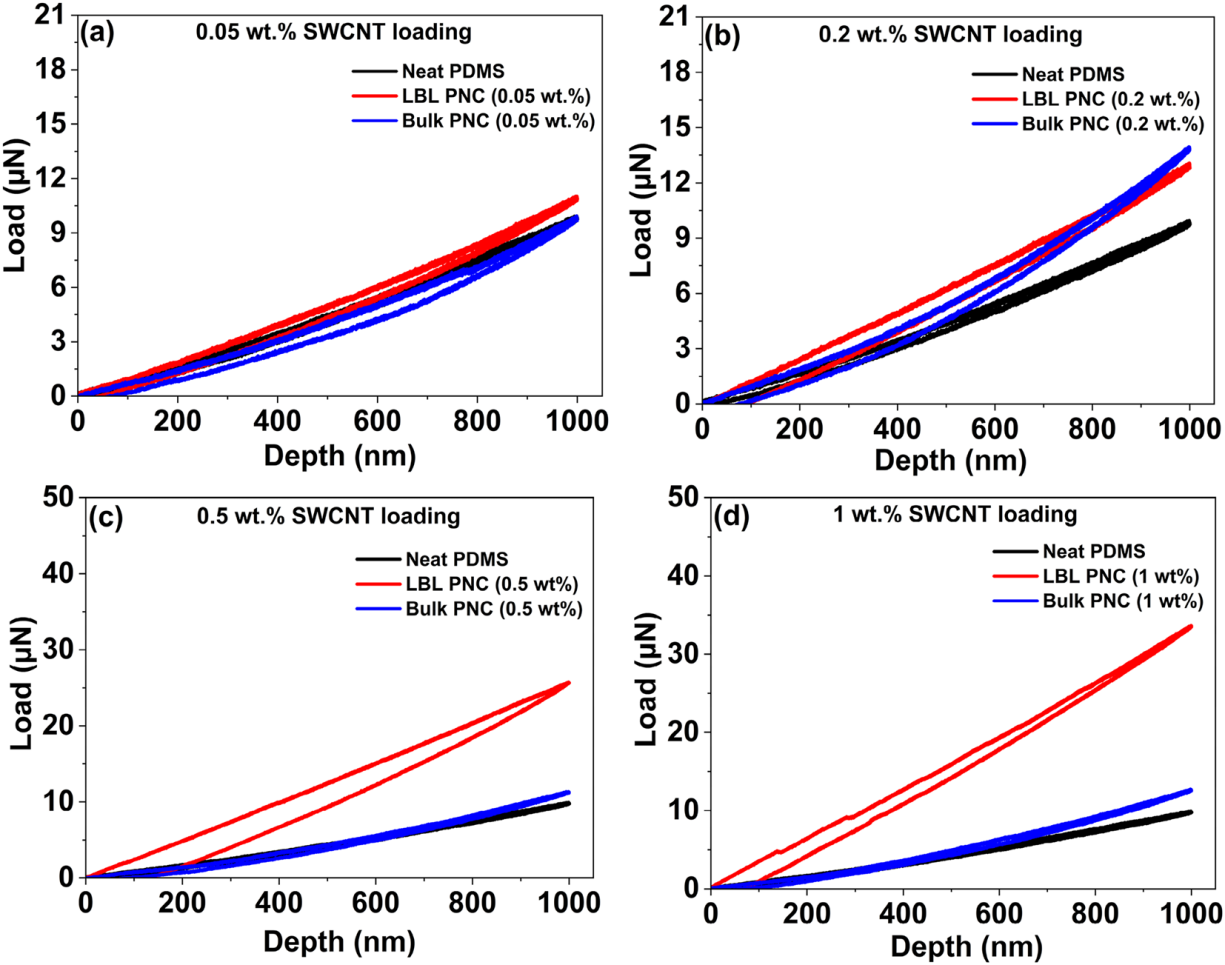
Comparative load–displacement curve from nanoindentation analysis for the samples with one representative curve from each analysis represented in terms of SWCNT loading wt% on PDMS (**a**) 0.05 wt%, (**b**) 0.2 wt%, (**c**) 0.5 wt%, and (**d**) 1 wt%. The plots reveal the 2.6X improvement in the resistance to indentation force by the LBL PNC (0.5 wt%).

Figure 9 is a comparative summary of the load–displacement analysis with one representative curve from each sample. Each sample was tested at over 50 points. Three representative curves for each sample are individually plotted and are depicted in Figs. S5 and S6 in the supplementary information. The improvement in the plastic deformation of the LBL PNCs over the bulk PNCs and neat PDMS is seen in Fig. 9a–d. From Fig. 9c, it is observed that LBL PNC (0.5 wt%) shows 2.6X improvement in the resistance to nanoindentation force compared to neat PDMS. In the loading–unloading curves, neat PDMS virtually exhibits total elastic recovery. It is significant to note that the area under the curves (representative of plastic work) for the LBL PNCs is significantly higher compared to their respective bulk PNCs and neat PDMS, in addition to the improvement in the amount of load the LBL PNCs can resist for an indentation depth of 1000 nm.

Hysteresis in nanoindentation refers to the phenomenon where the load–displacement curve during both loading and unloading cycles does not follow the same path. The hysteresis loop in nanoindentation is typically observed during the unloading phase of the experiment^73^. The loading phase involves gradually increasing the applied force, causing the indenter to penetrate the material. During unloading, the force is reduced, and the indenter is withdrawn from the material. Ideally, the unloading curve should follow the same path as the loading curve, but in many cases, there is a difference, and this difference is referred to as hysteresis^61,66,74–76^. The area under each curve is calculated and this hysteresis loss value is a representation of the plastic work (*W*_*p*_) that each composite material exhibits.

**Figure 10:**
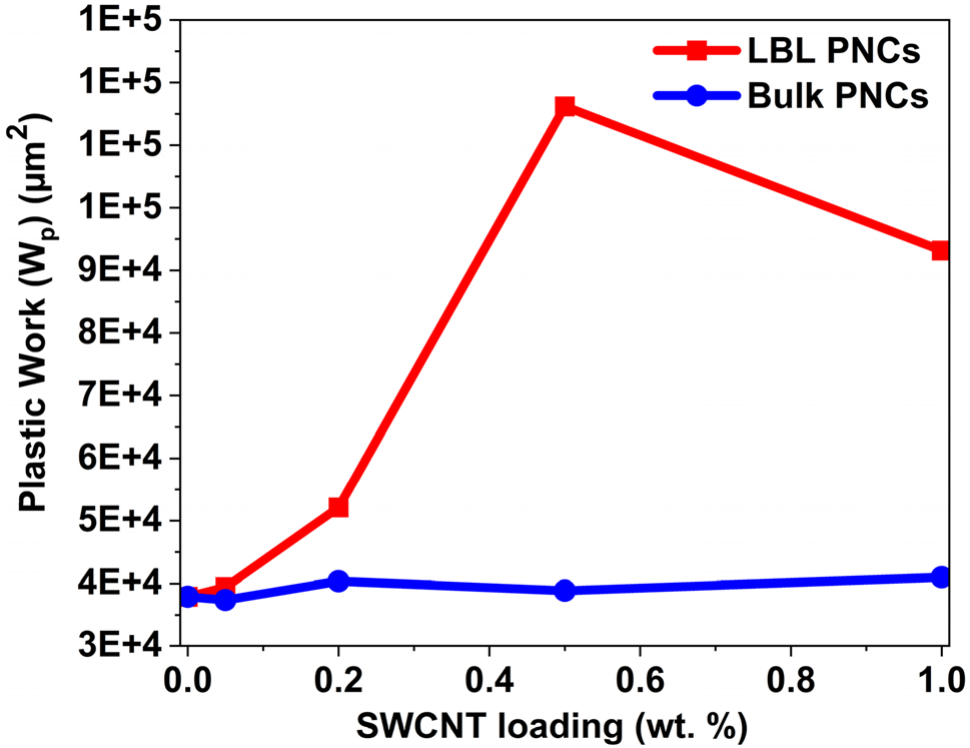
Plastic work undergone by each thin film sample upon nanoindentation with respect to SWCNT weight percentage in the composite. A 3.1X increase in the area under the curve for LBL PNC (0.5 wt%) with increasing SWCNT loading is observed from the plot.

Figure 10 represents the average area under the curve for the representative curves depicted in Figs. S5 and S6 in the supplementary information. It is a plot correlating the plastic work (*W*_*p*_) undergone by each sample with respect to the SWCNT weight loading percentage. The value is calculated by first calculating the area under the loading curve, which is the total work (*W*_*t*_). Next, the area under the unloading curve is calculated, which is the elastic work (*W*_*e*_) that the material exhibits. Then, the plastic work (*W*_*p*_) or the area under the curve is calculated using Eq. (1).

1 $${W}_{p}= {W}_{t}- {W}_{e}$$

As observed in Fig. 10, the area under the curve for each LBL PNC is superior to its bulk PNC counterpart. The plastic work of neat PDMS is lower compared to the composite thin films. Due to the presence of SWCNT on the top layer of the LBL PNCs, the tip is indenting more SWCNT than PDMS in LBL PNCs. However, as the presence of SWCNT in the bulk PNCs is not as well-distributed on the top surface as it is in the LBL PNCs, the plastic work of the bulk PNCs did not improve as significantly as it did in the LBL PNCs. LBL PNC (0.5 wt%) shows 3.1X improvement in the plastic work compared to neat PDMS. Plastic work (*W*_*p*_) continues to be better than the bulk PNCs and neat PDMS for 1 wt% SWCNT loading in the LBL PNCs. However, the *W*_*p*_ of LBL PNCs with 1 wt% SWCNT loading is less than that of 0.5 wt% SWCNT loading. This can be attributed to the effect of agglomeration amongst the SWCNT in the LBL structure at 1 wt% loading. The agglomeration of SWCNT in LBL PNC (1 wt%) is depicted in Figs. S1h and S2e in the supplementary information.

PDMS is a highly elastic material that can be stretched and compressed repeatedly without breaking or losing its mechanical properties. It has low stiffness, and therefore less or no plastic work is required to deform the material. The addition of SWCNT to PDMS increases the plastic work during indentation experiments. This is because SWCNT can reinforce the PDMS matrix, making it stiffer and more resistant to deformation under applied loads. Table 3 is a comparative summary of the references available on the nanoindentation of PDMS and PDMS-CNT composite films using a Berkovich tip. In this study, PDMS is subjected to a curing cycle of 70 °C and 4 h base: curing agent formulation ratio of 10: 1. This formulation is reported to produce neat PDMS with one of the lowest Young’s modulus (0.98 MPa) values reported for PDMS after the curing cycle^77^. Working with a low modulus PDMS clearly highlights the extent of enhancement that can be achieved in the mechanical properties of PDMS using the novel stack design at ultra-low-weight percentage reinforcement of sily-SWCNT. The results reveal the enhanced mechanical properties of the composite thin films fabricated in this study.

**Table 3:** Literature review table comparing the nanoindentation results on Sylgrad 184 PDMS and Sylgrad 184 PDMS-CNT based composites using Berkovich tip.

S. no	CNT wt% loading	PDMS thickness (µm)	PDMS base: curing agent ratio	Curing temperature and time	Depth of indentation (nm)	Maximum load (µN)	Plastic Work (W_p_)	Ref
1	0	3000	5:1	65 °C/1 h	5000 nm	80	191,260	^61^
2	0	45	15: 1	80 °C/ 8 h	5000 nm	57	163,811	^78^
3	0	200	10: 1	150 °C/15 min	1000 nm	13	8576	^26^
4	3				1000 nm	20	11,900	
5	0	175	10:1	70 °C/4 h	1000 nm	10	37,902	This work
6	0.05—Bulk	185				10	37,381	
7	0.2- Bulk	177				12	40,370	
8	0.5- Bulk	237				12	38,857	
9	1- Bulk	216				12	40,897	
10	0.05- LBL	179				11	39,404	
11	0.2- LBL	194				16	52,132	
12	0.5- LBL	316				35	116,197	
13	1- LBL	300				30	93,171	

#### Summary of reduced modulus (*E*_*r*_), young’s modulus (*E*), hardness (*H*), and contact stiffness (*S*_*c*_) maps

**Figure 11:**
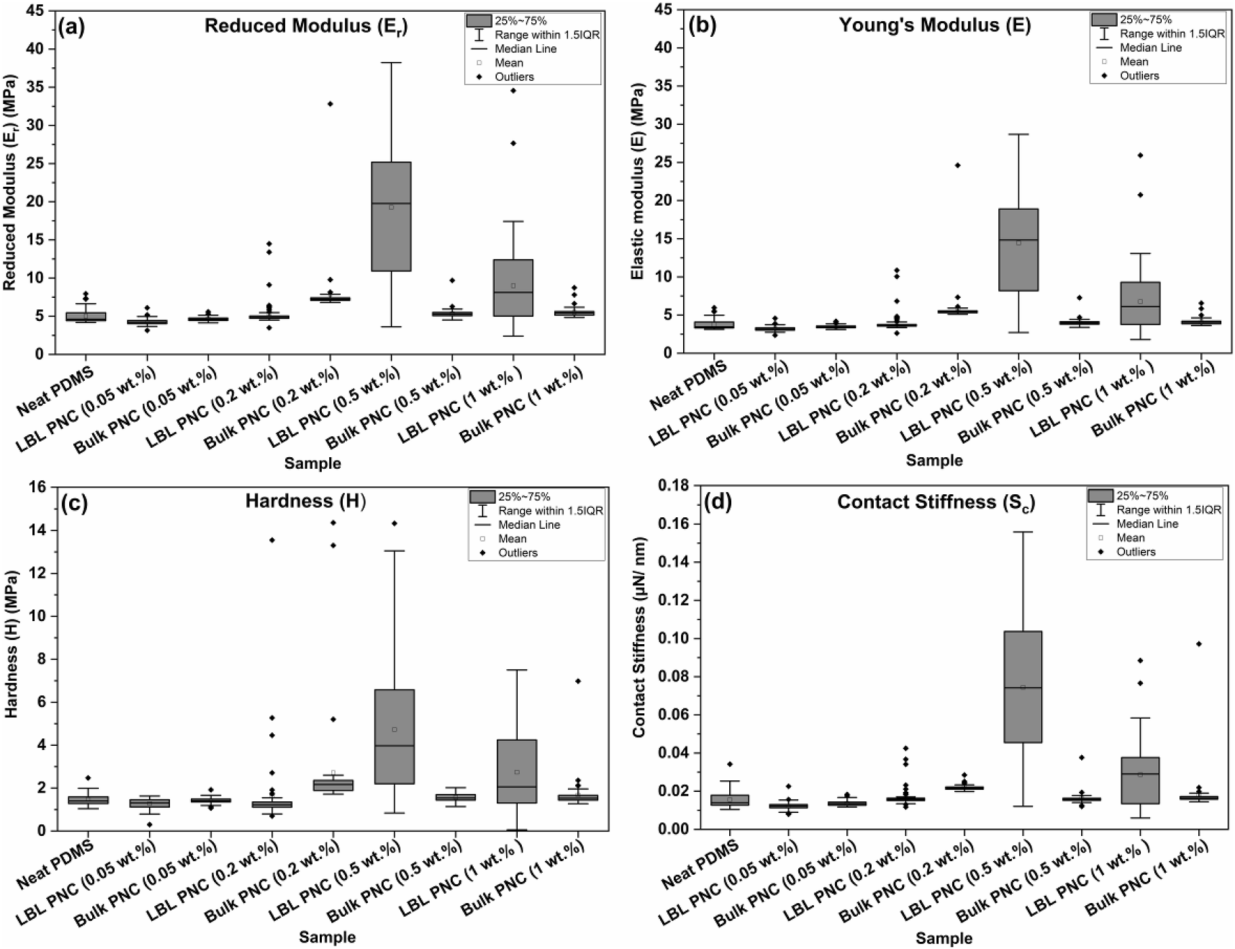
Box plots of data derived from nanoindentation analysis of thin films—(**a**) Reduced Modulus (*E*_*r*_), (**b**) Young’s Modulus (*E*), (**c**) Hardness (*H*) and (**d**) Contact Stiffness (*S*_*c*_). 3.8X, 2.5X and 4.6X improvement respectively in *E*, *H* and *S*_*c*_ values are represented in these figures due to the stack architecture in LBL PNC (0.5 wt%).

The reduced modulus (*E*_*r*_) and hardness (*H*) values are calculated for every single indentation point on the thin film sample. The Young’s Modulus (*E*) and contact stiffness (*S*_*c*_) of the samples are respectively calculated as discussed in S.2.1 and S.2.2 in the supplementary information. Figure 11a–d are box plots of reduced modulus (*E*_*r*_), Young’s Modulus (*E*), hardness (*H*), and contact stiffness (*S*_*c*_) respectively. The raw data for reduced modulus (*E*_*r*_) and hardness (*H*) from each point on the sample is available in Figs. S7–S10 in the supplementary information.

The reduced modulus (*E*_*r*_) of the thin film samples is calculated in accordance with Eqn. S1 in the supplementary information. Figure 11a reveals that the reduced modulus (*E*_*r*_) range of LBL PNC (0.5 wt%) is more than the values obtained from neat PDMS, bulk PNCs and the remaining LBL PNCs. The Young’s Modulus (*E*) values of each of the thin films are respectively calculated from the reduced modulus (*E*_*r*_) values using Eqn. S4 discussed in the supplementary information. The summary of the results is represented as a box plot in Fig. 11b. As supposed, the results follow the same trend as that of *E*_*r*_ values of the thin films. The hardness of the thin films is calculated using the following equation^65^.

2 $$H= \frac{P}{{A}_{c}}$$

where *P* is the applied load and *A*_*c*_ is the tip contact area during the indentation of the samples using the Berkovich tip. Based on the nanoindentation of each point on the thin film sample, hardness on each point is calculated using Eq. (2). The summary of the hardness of each thin film sample PNC (1 wt%) shows an improved range in the hardness values with respect to the rest of the samples under investigation. This is represented as a box plot in Fig. 11c. The hardness values of each of the thin film samples go in line with the Young’s Modulus (*E*) of the samples. LBL PNC (0.5 wt%) followed by LBL PNC (1 wt%) shows an improved range in the hardness values in comparison to the rest of the samples investigated.

The results from Fig. 11a–c reveal that the reduced moduli, elastic moduli, and hardness values of the LBL PNCs tend to possess a wider range of values when compared with their respective bulk PNCs. This can be witnessed in the case of the LBL PNCs with 0.5 wt% and 1 wt%. This shall be attributed to the increased presence of SWCNT on the top surface in the case of the LBL PNCs. The average values of Young’s Modulus (*E*), hardness (*H*), and contact stiffness (*S*_*c*_) of LBL PNC (0.5 wt%) have undergone significant increases, with respective improvements of 3.8X, 2.5X, and 4.6X. Due to the novel LBL-FP in LBL PNCs, the top surface of LBL PNCs tends to have a higher presence of SWCNT than the bulk PNCs. The bulk PNCs show results that are comparable to neat PDMS which is an indication that there is negligible improvement in the nanoindentation properties of the bulk composites in the lower weight percentage loadings (0.05–1 wt%). However, the LBL PNCs show significant improvement in indentation resistance analyzed through nanoindentation analysis at the same weight percentage loadings of SWCNT.

Contact stiffness (*S*_*c*_) of the thin film samples is calculated for each point of indentation using Eq. (5). From Fig. 11d, it is understood that the contact stiffness (*S*_*c*_) values of the thin films have significantly improved for the LBL PNCs with respect to their respective bulk PNCs and neat PDMS. In line with the trend observed in the reduced modulus (*E*_*r*_), Young’s Modulus (*E*), and hardness (*H*) values, the contact stiffness (*S*_*c*_) of LBL PNC (0.5 wt%) followed by the LBL PNC (1 wt%) tend to have a wider range of stiffness values with respect to the remaining samples under investigation.

### Delamination analysis

**Figure 12:**
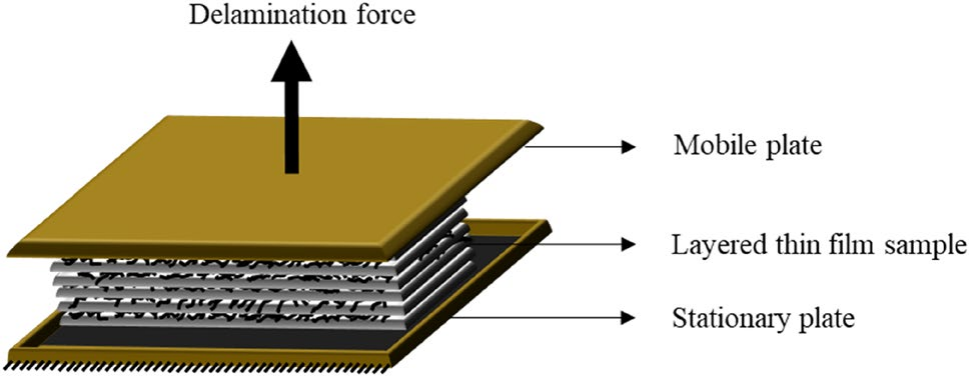
Schematic of the delamination set-up that was used to delaminate the multi-layered- thin film samples using a tensile force ramp. Image is represented for a schematic purpose and is not to scale.

In layered composite structures, delamination, i.e., interfacial fracture, is recognized as one of the most critical modes of failure^79–82^. Fracture along interfaces leads to loss of stiffness of the material/structure, load-carrying capacity reduction, and, ultimately, catastrophic events^82,83^. Delamination may be introduced at different stages of materials/structures' life-cycle due to different types of loading. For instance, for a laminated composite plate, the risk of delamination is due to trapped voids and air pockets^82,84^, the presence of release films and agents at an early stage of the production process^82,85^, machining or cutting^82,86^ and other actions such as mechanical, physical or chemical loading^82,87^. Until the very end of the catastrophic failure, flaws can be hard to identify, and, thus, the best way to avoid catastrophic events is to design ‘damage tolerant’ materials and structures, i.e., durable, and fail-safe^82,84,88^. A schematic of the process of delamination is shown in Fig. 12.

**Figure 13:**
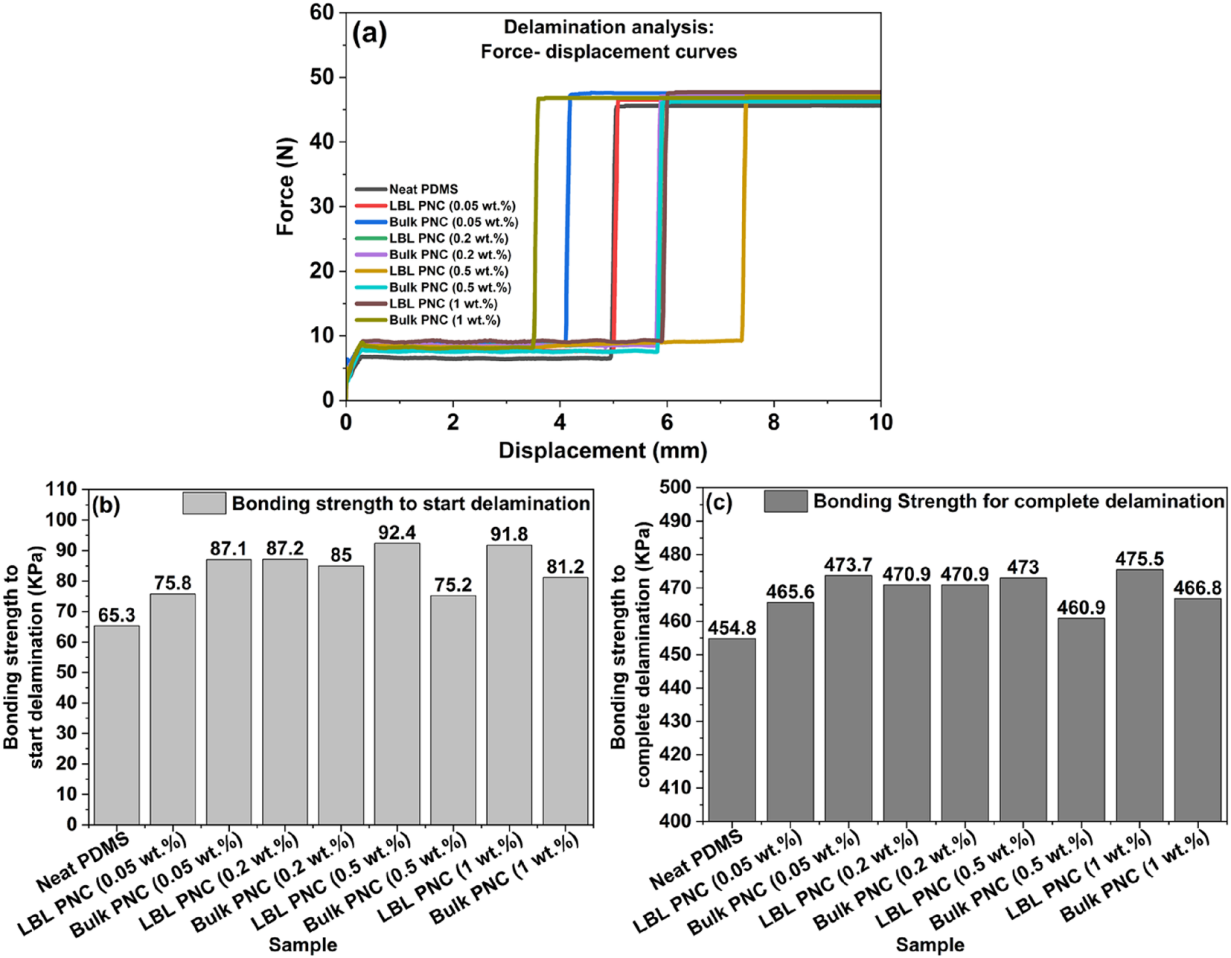
(**a**) Comparative delamination analysis of samples from force–displacement curves (**b**) Bonding strength of each thin film sample for initiation of delamination (**c**) Bonding strength of each thin film sample for completion of delamination. The bonding strength for initiation and completion of delamination shows 1.4X improvement in LBL PNC (0.5 wt%).

As shown in Fig. 12, the thin film sample is placed between the stationary and mobile plates of the delaminator using carbon tapes. The bottom plate is stationary, and the samples are subjected to delamination tensile force through the top plate. The top plate is subjected to a force ramp, and this instigates fracture in the sample leading to delamination of the thin film sample in the z-direction. The surface area of the delamination plates is 10^–4^ m^2^. Figure 13a shows the comparative force–displacement curves obtained from delamination analysis.

The force required to initiate and complete delamination is analyzed from the information in Fig. 13a. The bonding strength of each of the thin films is then calculated using the following equation.

3 $$Bonding\, strength \left(KPa\right)= \frac{Force (N)}{Area ({m}^{2})} \times {10}^{-3}$$

Using Eq. (3), the bonding strength that must be overcome in the thin film samples to initiate delamination and complete delamination is individually calculated and the results are represented in Fig. 15b and c. From Fig. 15b and c, it is understood that the bonding strength of each of the LBL PNCs is more than the bonding strength required to initiate and complete delamination in its respective bulk PNC counterpart. LBL PNC (0.5 wt%) shows the highest bonding strength to initiate delamination revealing a 1.4X improvement compared to neat PDMS. It is also observed that the bonding strength in both the bulk PNCs as well as the LBL PNCs is more than the required bonding strength in neat PDMS. It is worth noting that the force required to initiate delamination is more in the LBL PNCs with respect to its respective bulk PNC counterpart. According to a work by Mark A Eddings et al.^47^ the total bonding strength of Sylgrad 184 PDMS-based LBL fabricated multilayer system is reported to have a bonding strength of ~ 450 kPa for a 15:1 base: curing agent ratio. The results from our analysis go in line with this reported study.

**Figure 14:**
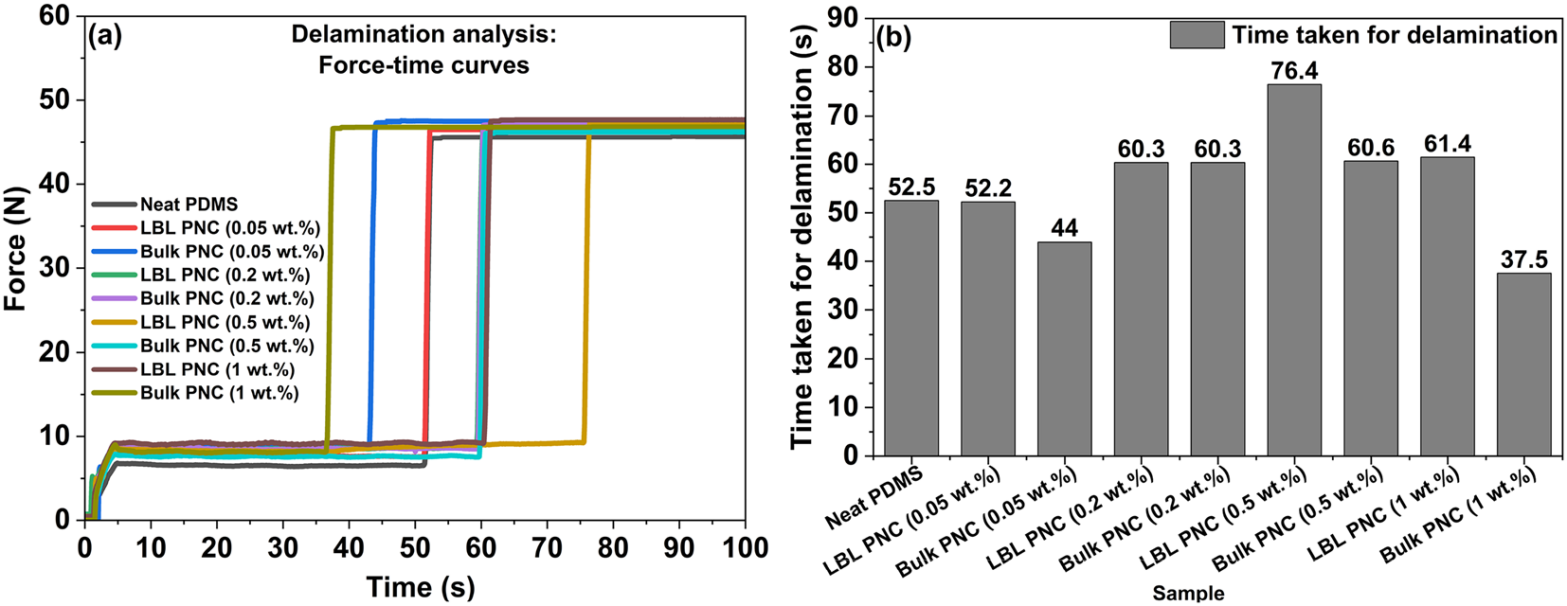
(**a**) Comparative delamination analysis of composite thin film samples from force–time curves, (**b**) Total time taken for delamination of the thin film samples. Figure 14(**b**) shows a 1.5X improvement in the time taken for delamination for LBL PNC (0.5 wt%).

Figure 14a represents the force–time curves that were plotted and analyzed to estimate the total time required for the complete delamination of the thin film samples. The observation made in the force–displacement plot in Fig. 13a is also seen in the time taken to completely delaminate the thin film samples in Fig. 14a. Figure 14b is a representation of the time taken to completely delaminate the thin film samples. From Fig. 14b, it is observed that the time taken for the delamination of LBL PNCs is more than the time taken for the delamination of bulk PNCs. LBL PNC (0.5 wt%) takes 1.5X more time to fully delaminate compared to neat PDMS. This can be attributed to the crack propagation mechanics in the samples.

**Figure 15:**
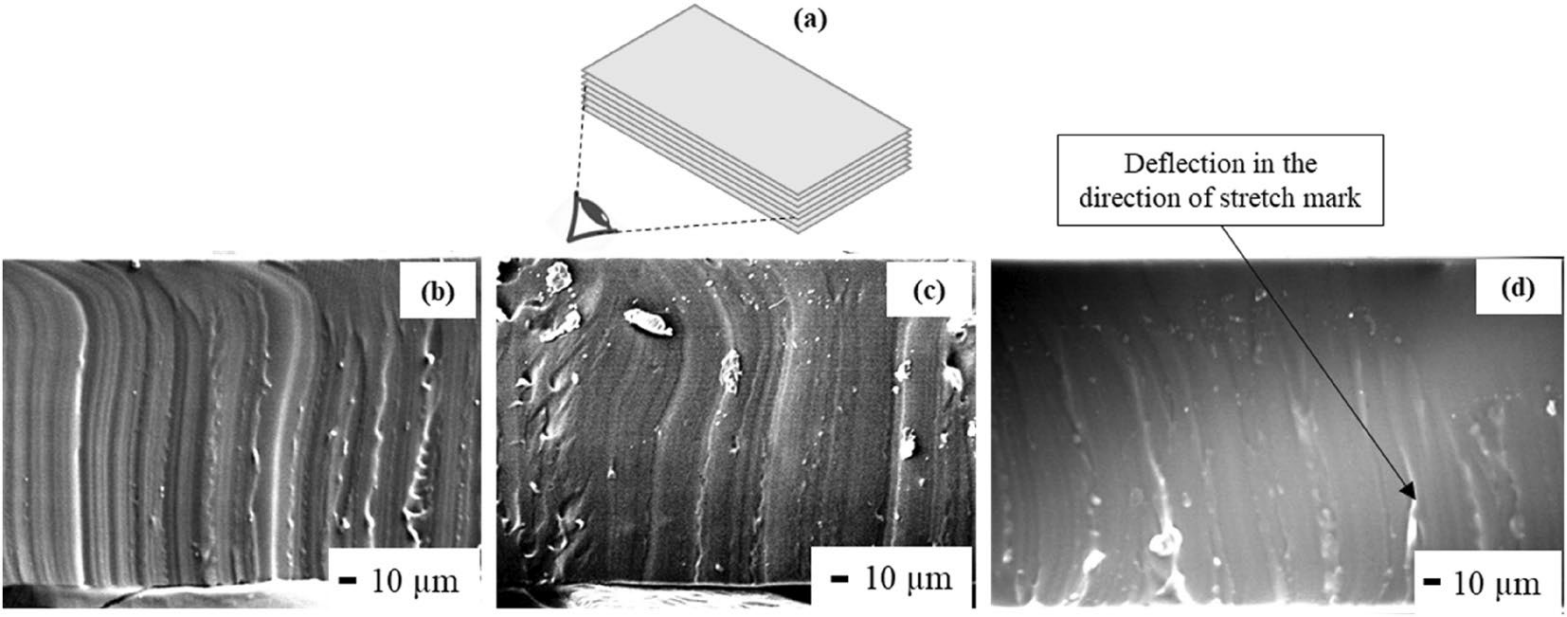
Comparative FESEM analysis of the layer-by-layer fabricated thin film samples: (**a**) viewing angle of the delaminated samples, (**b**) neat PDMS, (**c**) bulk PNC (0.5 wt%) and (**d**) LBL PNC (0.5 wt%). The figures show stretch marks after tensile delamination test on the elastic PDMS based films representing the crack propagation pattern across the cross section of the thin films.

The cross-section of the samples after delamination tests were analysed using FESEM to check the morphology after the experiment. Figure 15 comparatively represents the cross-section of the samples after delamination. Figure 15a is a schematic of the viewing angle of the cross-section of the samples in the FESEM equipment after delamination test.

From Fig. 15, it is understood that due to the elastic nature of PDMS, during delamination tests, the thin film samples stretch along the direction of force and de-bonds when the maximum force is applied. As a result of this, the samples were found to have stretch marks along the direction of force after delamination test. All the samples were tested within 20 min after the completion of delamination tests, and it is assumed that the samples elastically recovered to a major extent before they were subjected to FESEM analysis.

The presence of continuous vertical stretch marks along the full thickness of the sample is prominent in neat PDMS (Fig. 15b). In bulk PNC (0.5 wt%) (Fig. 15c), vertical stretch marks along the full length of the sample are observed. It can be noted that the stretch marks are not as noticeable as they are in neat PDMS, and the stretch marks are also not fully vertical. This indicates the discontinuity in the matrix due to presence of SWCNT that diverts the direction of stretching in the sample. In line with this trend, the stretch marks in LBL PNC (0.5 wt%) (Fig. 15d) are not fully seen through the thickness of the sample. They are seen only through 50–75% of the thickness of the sample. It can also be noted that the stretch marks are slanting in nature and take a criss-cross path to propagate through the thickness of the sample. Due to the presence of SWCNT in every alternating layer in LBL PNC, the stretch marks get redirected indicating the prominence of SWCNT along the thickness of the sample. This nature of the design has increased both the debonding strength and time for LBL PNCs.

**Figure 16:**
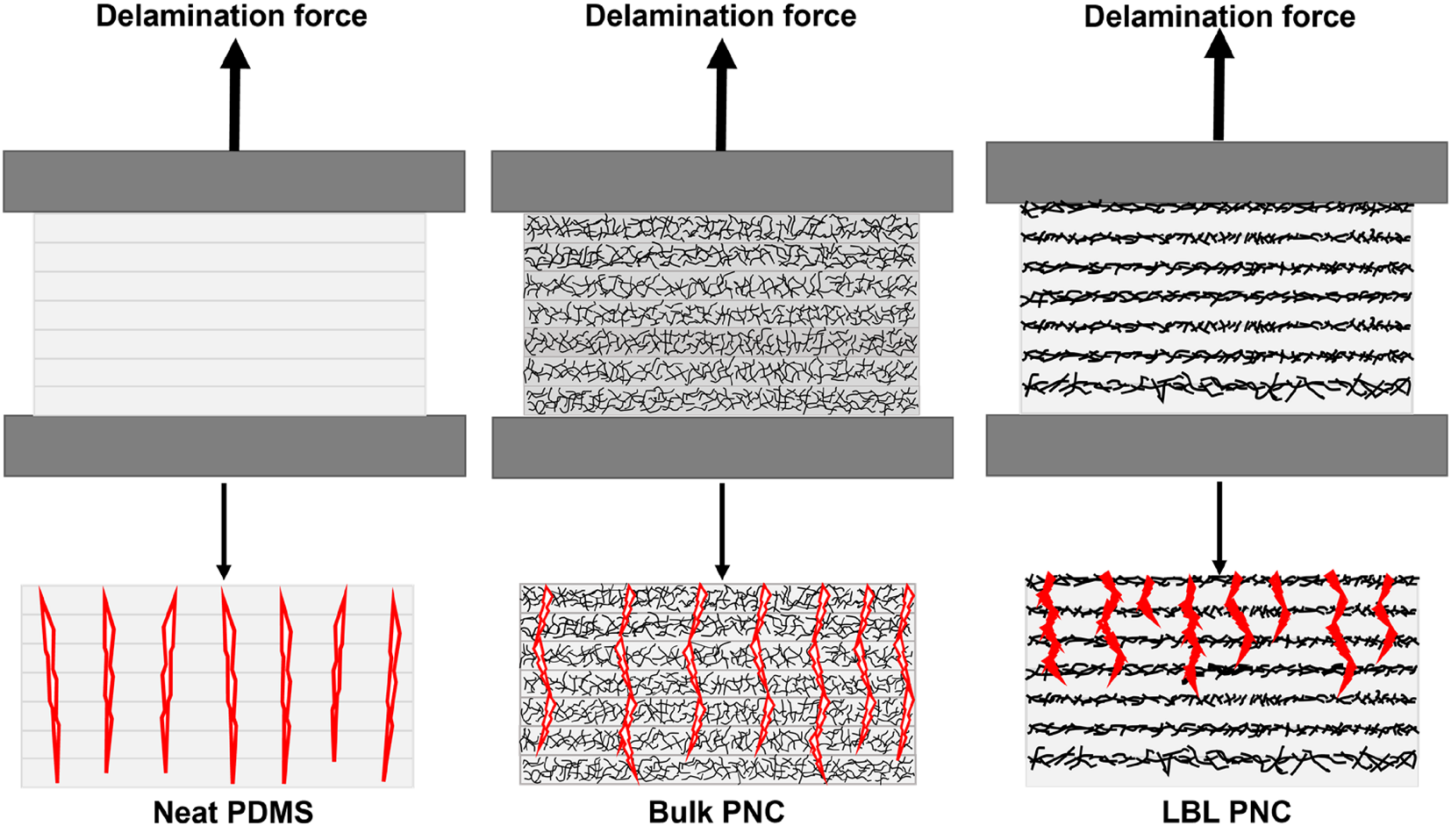
Comparative schematic of delamination force and crack propagation in LBL stacked films. The figure is a hypothesis of the possible crack propagation across the cross section of the thin films during delamination analysis at a given time.

Based on the results in Figs. 13a–c, 14a,b and 15b–d, the crack propagation mechanism leading to fracture in neat PDMS, LBL PNCs, and bulk PNCs is proposed as a schematic in Fig. 16. As indicated in Fig. 16, due to the resistance presented by a dense SWCNT layer in every alternate layer in the LBL PNCs, continuous crack propagation is resisted by this architecture. This is the reason for comparatively higher bonding strength and longer time taken for delamination of LBL PNCs.

### Dynamic mechanical analysis

One of the fundamental measurements of DMA TAQ800 equipment is the bulk stiffness (*k*) of the material, which is a geometry-dependent property of a material. The bulk stiffness (*k*) of the thin film samples is calculated by the DMA unit using the following equation^89^

4 $$k= \frac{Force\, applied\, to \,sample \left(N\right)}{Amplitude\, of\, deformation \left(m\right)}$$

As discussed in a study by R. Seghir et al.^90^ the stiffness of PDMS can be tuned by modifying three main parameters: (i) the cross-linker agent concentration, (ii) the curing temperature, and (iii) the curing time. Other parameters also affect stiffness, e.g., film thickness, micromachined object dimensions, and the loading strain rate.

**Figure 17:**
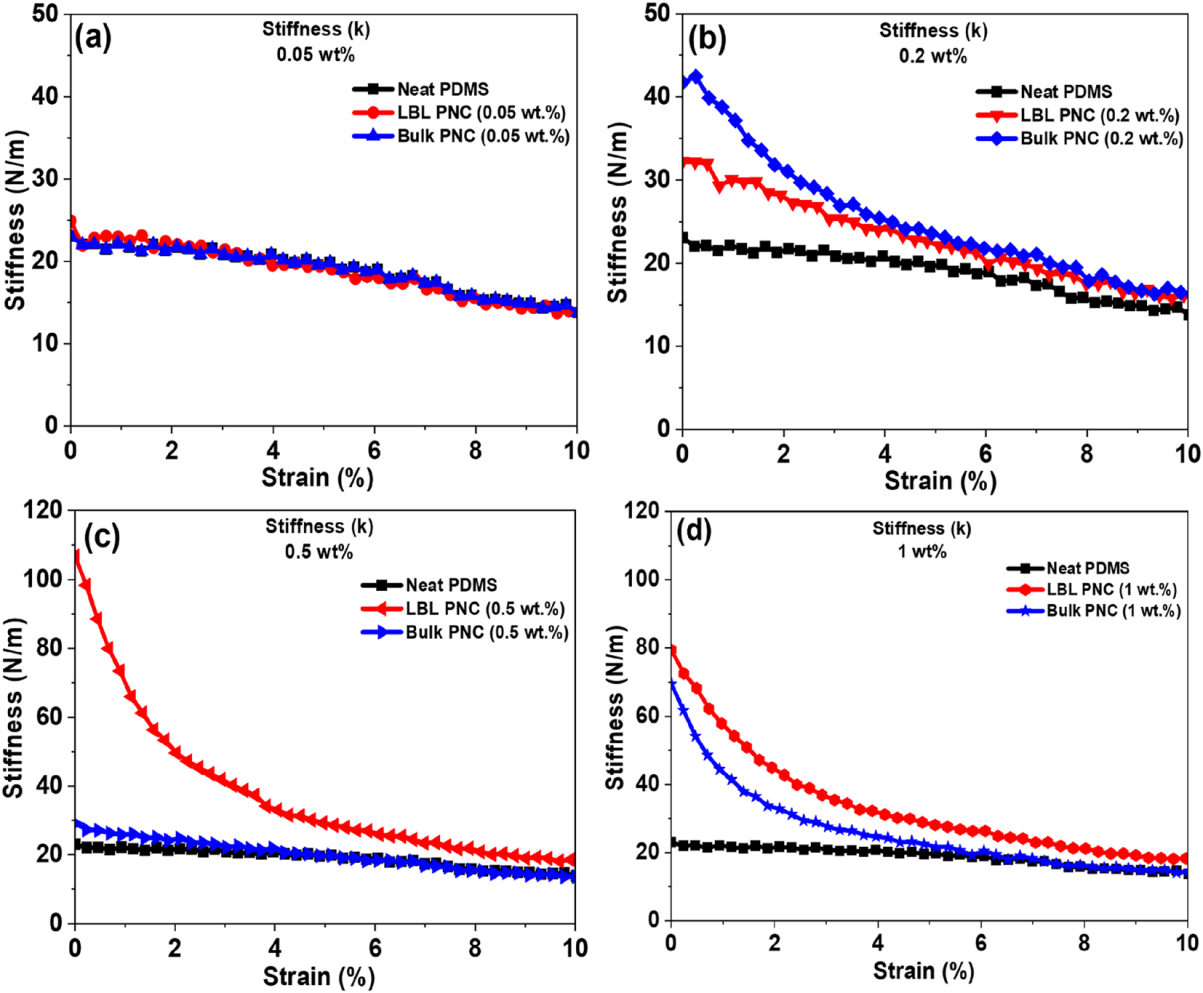
Comparative bulk stiffness (*k*) of the samples over a strain sweep at 20 Hz. There is 3.7X improvement in *k* values for LBL PNC (0.5 wt%) (Fig. 17(**c**)).

Figure 17a is a comparative plot of the bulk stiffness (*k*) of the thin film samples over a strain sweep investigated at a constant frequency of 20 Hz. It is observed that the stiffness values of the LBL PNCs with 0.5 wt% and 1 wt% SWCNT loading is superior to their respective bulk PNC counterparts by 3.7X and 1.2X respectively. Like the previous trends observed in the other mechanical studies, each of the LBL PNCs exhibits improved results with respect to its bulk PNC counterpart. In line with the contact stiffness (*S*_*c*_) results discussed in Section “Summary of reduced modulus (Er), young’s modulus (E), hardness (H), and contact stiffness (Sc) maps” (Fig. 11d), the bulk stiffness (*k*) values calculated from DMA tend to follow the same trend. The bulk stiffness (*k*) value of the LBL PNC with 0.5 wt% SWCNT loading is found to be 4.6X more than the *k* values of the other samples.

It is also interesting to note that for lower strain values (< 5%), the *k* values are significantly higher than the *k* values obtained at larger strains. This trend holds true for composite samples (both LBL and bulk PNCs) across the four different weight percentages and is depicted in the plots shown in Fig. 17. This may be attributed to the process where during tensile deformations the SWCNT in the film is stretched farther apart and the thickness of the film starts to decrease to maintain the volume of the film intact during elongation. It can also be hypothesized that during tensile deformations, the SWCNT bundles distributed in the film may break into smaller fractured CNTs. Due to the thinning of sample and the possible breaking of SWCNT bundles into smaller dimensions, the stiffness of the composite thin films decreases with increasing strain.

Storage modulus (*E’*) is a measure of the stored energy in a viscoelastic material and represents the elastic portion of the material. It is represented by the following equation^89^.

5 $${E}^{\prime}= \frac{Stress (\sigma )}{Strain (\varepsilon )} \times {\text{cos}}\delta ,$$

where *δ* is the phase angle between the sinusoidal strain (*ε*) applied to the sample at 20 Hz frequency and measured stress (*σ*).

**Figure 18:**
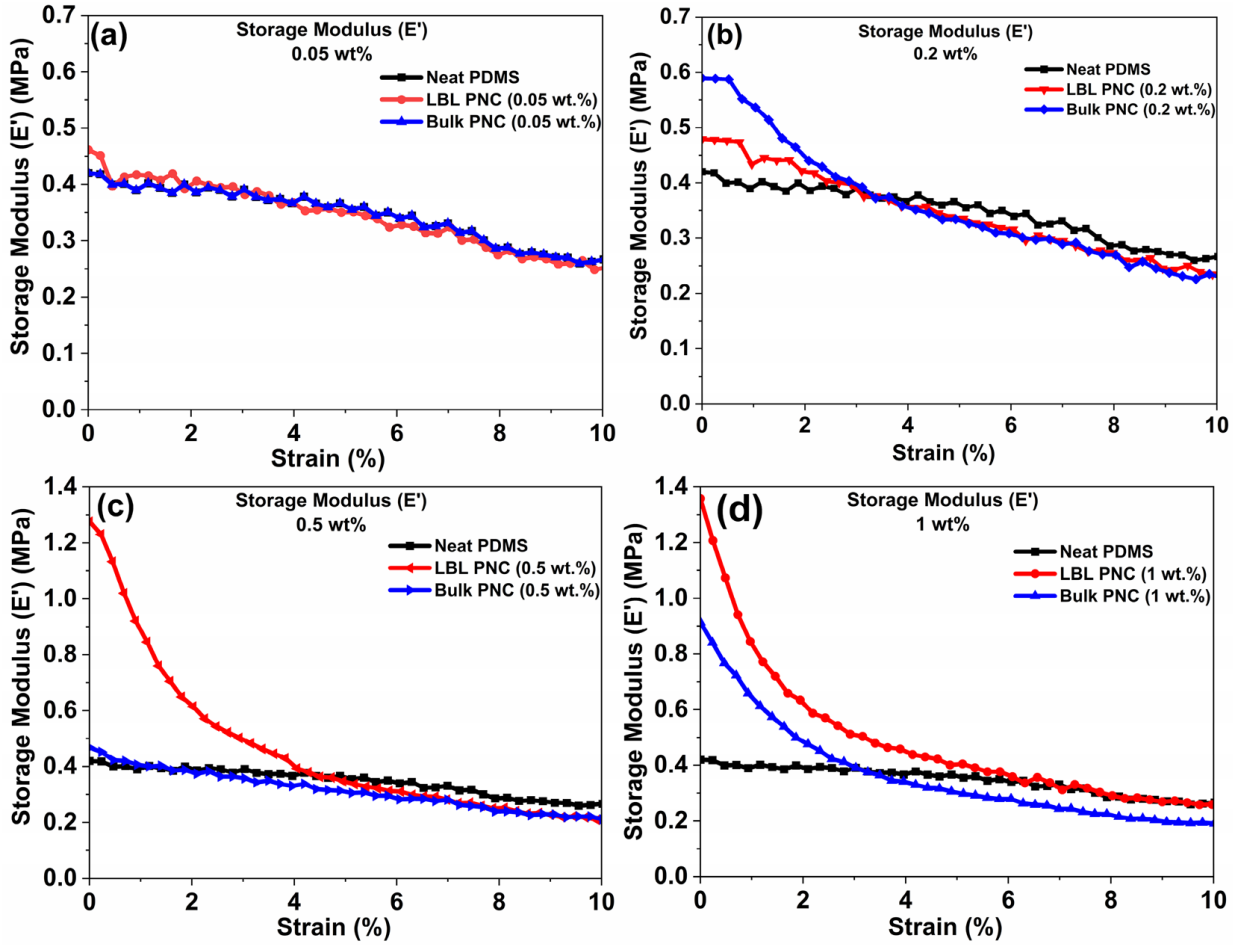
Comparative storage modulus (*E’*) of the samples over a strain sweep at 20 Hz. The plots reveal the 3.X improvement in *E’* values of LBL PNC (0.5 wt%) (Fig. 18(**c**)).

Figure 18 is a comparative plot of the storage modulus (*E’*) of the thin film samples over a strain sweep at 20 Hz. It is noteworthy that the storage modulus of the LBL PNC (0.5 wt%) is superior by 3X times compared to the storage modulus of the other composite thin films under study. The result indicates the favourable effect of LBL curing and the promising load-bearing effect of SWCNT layers on the overall enhancement of the stiffer SWCNT-PDMS thin film at 0.5 wt% SWCNT loading^51^.

The trend is in coherence with the previously discussed results indicating that the mechanical reliability of 0.5 wt% SWCNT in the LBL PNC is superior to the other composite thin films. When PDMS/SWCNT composites are subjected to increasing strain during DMA testing, the storage modulus typically decreases. This is because at higher strains, the PDMS/SWCNT chains are stretched further apart and the composite becomes less compact, which results in a reduction in its ability to store elastic energy. Additionally, at higher strains, some of the SWCNT may get detached from the PDMS matrix (loss of adhesion between SWCNT and PDMS), which further contributes to the reduction in the storage modulus. Table 4 is a comparative summary of the storage modulus values estimated through DMA studies for PDMS and PDMS-CNT-based composite thin film samples. It can be observed from this table that the LBL-FP proposed in this study can produce stiffer composite thin film structures with enhanced mechanical properties along the thickness of the film which is crucial to delay fracture and failure of these structures along the direction in which their dimension is confined. (z-axis).

**Table 4:** Literature review table of storage modulus (*E’*) of Sylgrad 184 PDMS and Sylgrad 184 PDMS-CNT based composites.

S. no	CNT wt% loading	PDMS dimensions (l × w × t) (mm^3^)	PDMS base: curing agent ratio	Curing temperature and time	Storage modulus (E’) at 25 °C (MPa)	Test method	Ref
1	0	20 × 8.5 × 1	10:1	60 °C/16 h	1.8	1 Hz Freq., 25–40 °C Temp sweep at 1 °C/min ramp	^6^
2	0	20 × 2 × 0.2	10:1	150 °C/15 min	1.8	20 Hz Freq., Strain sweep at 0.001 N tension trigger	^26^
3	0				1.3	40 Hz Freq., Strain sweep at 0.001 N tension trigger	
4	1				1.8		
5	2				2.6		
6	4				2.7		
7	0	35 × 14 × 3.8	10:1	65 °C/4 h	2.14	Room temp., Freq. sweep 0.01 to 80 Hz, Freq. = 20 Hz	^39^
8	0				2.5	Room temp., Freq = 80 Hz, ageing time = 4 h	
9	0			65 °C/10 h	2.7		
10	0	t = 1.35	10:1	50 °C/24 h	3.10	1 Hz Freq., − 100 °C to 200 °C Temp. sweep at 5 °C/min	^51^
11	2	t = 0.3	10:1	60 °C/5 h	0.5	1 Hz Freq., 0 to 20% Strain sweep	^91^
12	0	t = 3.8	10:1	60 °C/1 h	1	1 Hz Freq., − 140 °C to 150 °C Temp. sweep at 3 °C/min; applied strain- 0.05%	^92^
13	0	7.5 × 3.5 × 0.18	10:1	70 °C/4 h	0.42	20 Hz Freq., 0.1 to 1000 µm strain sweep	This work
14	0.05—Bulk				0.42		
15	0.2- Bulk				0.58		
16	0.5- Bulk				0.47		
17	1- Bulk				1.21		
18	0.05- LBL				0.46		
19	0.2- LBL				0.48		
20	0.5- LBL				1.28		
21	1- LBL				0.91		

**Figure 19:**
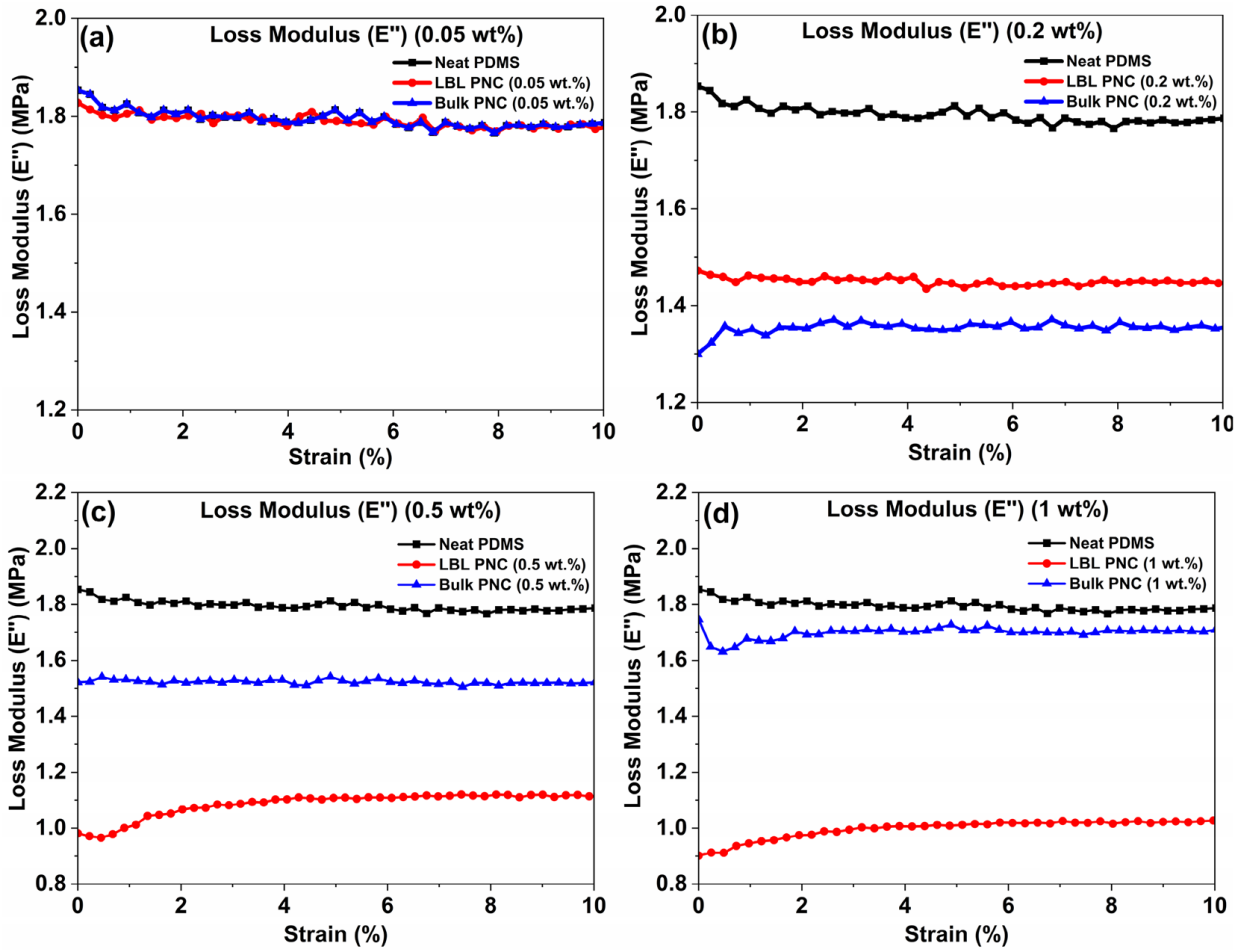
Comparative loss modulus (*E’’*) of the samples over a strain sweep at 20 Hz.

**Figure 20:**
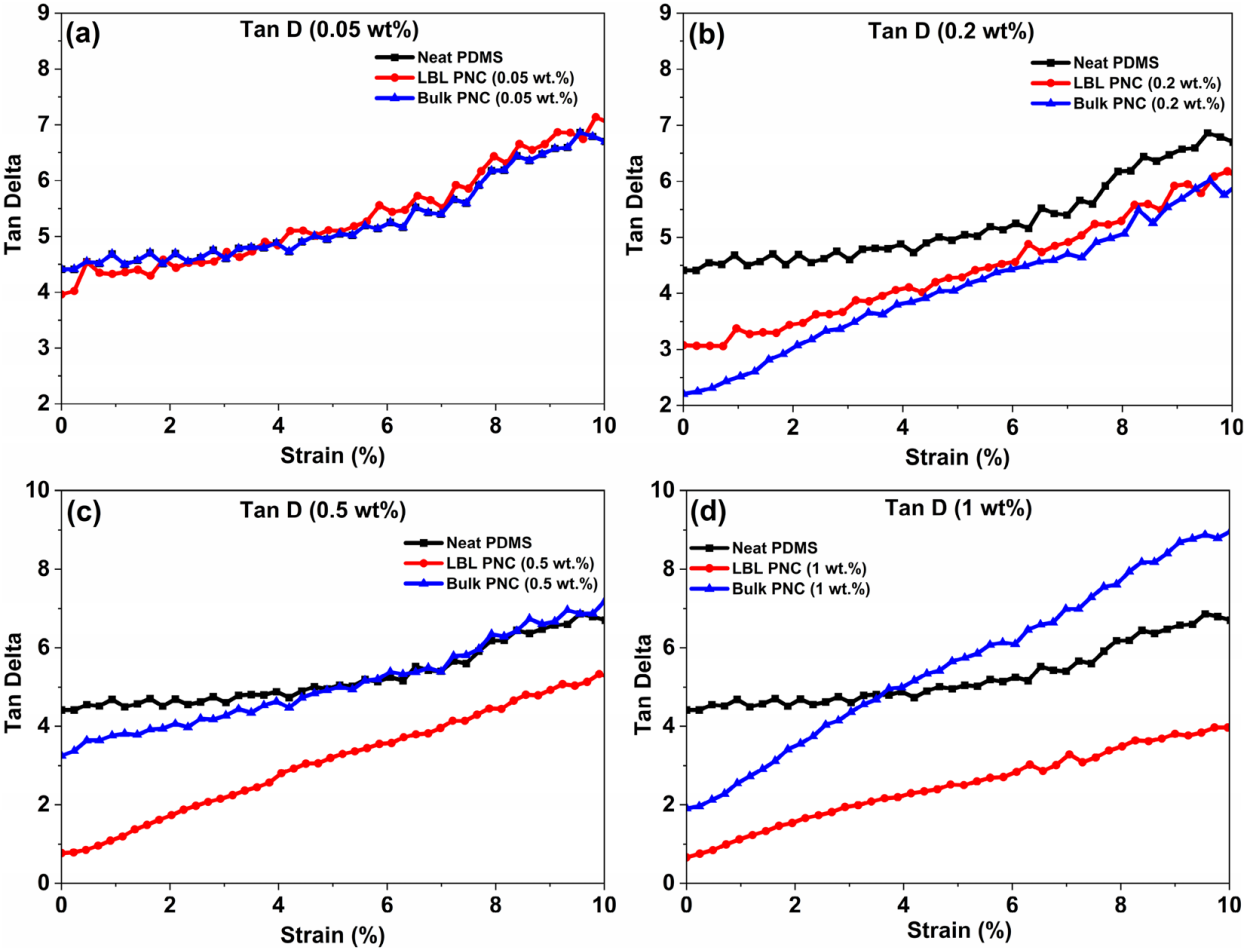
Comparative Tan delta values of the samples over a strain sweep at 20 Hz.

The graphical representation of the loss modulus magnitude (disregarding phase lag between storage and loss modulus measurements by the instrument) and Tan delta of the test structures is illustrated in Figs. 19 and 20. DMA is employed to quantify stiffness and damping, with these parameters being reported as modulus and Tan delta. The application of a sinusoidal force allows the modulus to be expressed as two components: the in-phase component known as the storage modulus (*E’*), and the out-of-phase component termed the loss modulus (*E”*). The storage modulus (*E’*) characterizes the elastic energy stored in a material, while the loss modulus (*E”*) reflects the material's viscous response^89^.

When the storage modulus surpasses the loss modulus, the material is predominantly elastic. Conversely, if the loss modulus exceeds the storage modulus, the material exhibits a predominant viscous behavior, dissipating more energy than it can store—akin to a flowing liquid. Examination of Figs. 18 and 19 reveals that for LBL PNC (0.5 wt%) and LBL PNC (1 wt%), the storage modulus (*E’*) is greater than the loss modulus (*E''*). This observation indicates a substantial increase in the elastic nature of the composite material due to the reinforcement of sily-SWCNT in the LBL PNC architecture, even at ultra-low weight percentage loadings^89^.

The ratio of the loss modulus to the storage modulus is defined as the damping factor or loss factor and denoted as tan δ. Tan δ indicates the relative degree of energy dissipation or damping of the material. For example, a material with a tan δ > 1 will exhibit more damping than a material with a tan δ < 1, because the loss modulus is greater than the storage modulus in the former, which means the energy dissipating, viscous mechanisms will have a greater influence on the final properties of the material^89^. From Fig. 20, it can be understood that only LBL PNC (0.5 wt%) and LBL PNC (1 wt%) samples have tan δ < 1 indicating the efficient reinforcement of sily-SWCNT in PDMS improving the elastic properties over the viscous properties in these two samples.

### Summary of the results from mechanical characterization

The multilayered samples fabricated in this study were extensively subjected to three different mechanical testing methods. The nanoindentation test investigated the local mechanics of the composites at a nanoscale using a perpendicular, compressive, indentation force on the surface. Delamination analysis was used to understand the macro scale static tensile properties across the thickness of the composites. Dynamic mechanical analysis was employed to understand the dynamic tensile properties of the composite across all three dimensions of the thin films. All methods coherently revealed that the LBL PNCs outperformed their respective bulk PNC counterparts across all weight percentage loadings of SWCNT.

Table 5 summarizes the mechanical characterization of composite thin film samples obtained through the novel LBL-FP. The number of layers and total thickness can be controlled by optimizing the spinning parameters during fabrication. Silylation of SWCNT makes the filler hydrophobic, promoting adhesion and dispersion with the hydrophobic PDMS matrix. The covalent chemical bond between SWCNT and PDMS enhances the connection, enabling multifunctional property enhancement such as plastic work (*W*_*p*_), Young’s Modulus (*E*), hardness (*H*), contact stiffness (*S*_*c*_), bonding strength, bulk stiffness (*k*), and storage modulus (*E’*).

**Table 5:** Consolidated summary of the mechanical characterization results from the LBL fabricated composite thin films.

S. no	Sample name	Nanoindentation analysis (50–80 points on each sample)						Delamination analysis- average of results from 3 samples			DMA analysis- average of results from 3 samples	
		Maximum load (µN)	Plastic work (*W*_*p*_)	Mean *E*_*r*_ (MPa)	Mean *E* (MPa)	Mean *H* (MPa)	Mean *S*_*c*_ (µN/ nm)	Bond Strength- to initiate delamination (KPa)	Bond Strength- to complete delamination (KPa)	Time taken for complete delamination (s)	*k* at 0% strain (N/m)	*E’* at 0% strain (MPa)
1	Neat PDMS	9.8	37,902	5	4	2	0.016	65.3	454.8	52.52	23.05	0.42
2	LBL PNC (0.05 wt%)	9.8	39,404	4	3	1	0.012	75.8	465.6	52.22	24.92	0.46
3	Bulk PNC (0.05 wt%)	9.8	37,381	5	4	1	0.014	87.1	473.7	44.03	23.05	0.42
4	LBL PNC (0.2 wt%)	12.9	52,132	5	4	2	0.017	87.2	470.9	60.33	32.27	0.48
5	Bulk PNC (0.2 wt%)	13.86	40,370	8	6	3	0.022	85	470.9	60.33	41.74	0.58
6	LBL PNC (0.5 wt%)	25.7	116,197	20	15	5	0.074	92.4	473	76.42	106.81	1.28
7	Bulk PNC (0.5 wt%)	11.29	38,857	5	4	2	0.016	75.2	460.9	60.63	29.16	0.47
8	LBL PNC (1 wt%)	33.6	93,171	9	7	3	0.029	91.8	475.5	61.43	79.42	1.36
9	Bulk PNC (1 wt%)	12.58	40,897	5	4	2	0.018	81.2	466.8	37.54	69.43	0.93

LBL PNC (0.5 wt%) exhibits superior mechanical properties, overcoming challenges of SWCNT dispersion in PDMS. With respect to neat PDMS, LBL PNC (0.5 wt%) tends to possess 2.6X improved resistance to nanoindentation force and better viscoelastic behavior with an improvement in plastic work (*W*_*p*_) by ~ 3.1X. The mean Young’s Modulus (*E*), mean hardness (*H*), and mean contact stiffness (*S*_*c*_) have improved by 3.8X, 2.5X, and 4.6X respectively. The bond strength (B.S.) is observed to have improved by ~ 1.2X and the time taken for delamination has increased by 1.5X. The bulk stiffness (*k*) of the samples has increased by 4.6X along with a 3X improvement in storage modulus (*E’*).

## Conclusions

In this work, a novel multilayered structure is reported with alternating layers of PDMS and SWCNT (LBL PNCs). This work is the first of its kind to evaluate the mechanical properties of this novel multilayered design. The three different mechanical characterization techniques used in this study intricately reveal the nano to macro level mechanics of flexible composites. This work is also noteworthy in terms of evaluating properties of silylated SWCNT reinforced PDMS composites. The multilayered design has specifically improved the properties of the thin films along the direction of its thickness (z-direction) (revealed through nanoindentation and delamination analyses) and has improved the overall three-dimensional mechanical properties (revealed through dynamic mechanical analyses). This study reveals that the mechanical properties of the multilayered design in LBL PNCs significantly improves even at ultra-low weight percentage loadings of silylated SWCNT. The significant improvement in the mechanics of the composite can be attributed to the novel multilayered structure and the better chemical adhesion between silylated SWCNT and PDMS.

While the mechanical characteristics of the LBL PNCs were deliberately enhanced throughout the film's thickness via an innovative multilayered configuration, this arrangement results in non-uniform mechanical properties for the composite. The arrangement of SWCNT within each alternating layer is achieved through self-assembly and does not exhibit uniformity across the entire composite. Thorough mechanical analysis is necessary to gain a comprehensive understanding of the implications of this non-uniformity. Additionally, conducting in-situ tests that involve subjecting LBL PNCs to mechanical deformation while simultaneously performing microscopic analysis is a prospective avenue for future research, offering significant potential for unveiling the unique mechanics of LBL PNCs.

This novel multi-layered design shows promising mechanical performance at low SWCNT loading, with potential applications in soft and lightweight products such as wearable strain sensors, flexible circuits, and lab-on-a-chip devices. In the near future, there is potential for conducting additional research on the impact of changes in stiffness on the functional properties of PDMS such as rupture limit, Poisson's ratio, and wetting contact angle. Such findings could prove beneficial to technology application-focused groups that employ PDMS-based polymers in their research. The results would offer a deeper understanding of the mechanical properties, deformation behavior, and surface properties of the material, which could inform the selection and design of materials for application-specific optimal performance.

### Supplementary Information

Supplementary Information.

## Data Availability

The datasets generated during and/or analysed during the current study are available from the corresponding author on reasonable request.
