# Supplementary material for: Enhancement of the mechanical properties in ultra-low weight SWCNT sandwiched PDMS composites using a novel stacked architecture
Source: Sci Rep. 2024 Feb 23;14:4487. doi: 10.1038/s41598-024-54631-7 (PMC10891152; doi:10.1038/s41598-024-54631-7)
Supplement: Supplementary file 1 — Supplementary Information. [file 41598_2024_54631_MOESM1_ESM.docx]

**Enhancement of the Mechanical Properties in Ultra-Low Weight SWCNT Sandwiched PDMS Composites Using a Novel Stacked Architecture**

Pavithra Ananthasubramanian^1^, Rahul Sahay^1^, Nagarajan Raghavan^1^*

^1^nano- Macro Reliability Laboratory (nMRL), Engineering Product Development (EPD) Pillar, Singapore University of Technology and Design, 8 Somapah Road, 487372, Singapore

*Corresponding author’s email address: [nagarajan@sutd.edu.sg](mailto:nagarajan@sutd.edu.sg)

**SUPPLEMENTARY INFORMATION**

**S1. Top-surface analysis: LBL PNCs vs. Bulk PNCs**

The top surface of neat PDMS and all the composite samples were imaged using FESEM at 5000X magnification. Fig. S1 is a comparative representation of the FESEM imaging of the composite samples. The LBL PNCs have CNT on their top layer, and it is clearly visible in the FESEM images for 0.5 wt% and 1 wt% samples (Fig. S1 (f) & (h)). As anticipated, the nanotube distribution on the surface of the LBL PNC composites increases with increasing weight percentage of SWCNT loadings in the composite. The curled-up edges from the samples revealing the cross-section were also imaged to correlate the images in Fig. S2 with the FESEM images in Fig. 6 and 7 in the main text. Fig. S2 is a summary of the cross-section of the LBL PNC samples imaged from the top view.

**
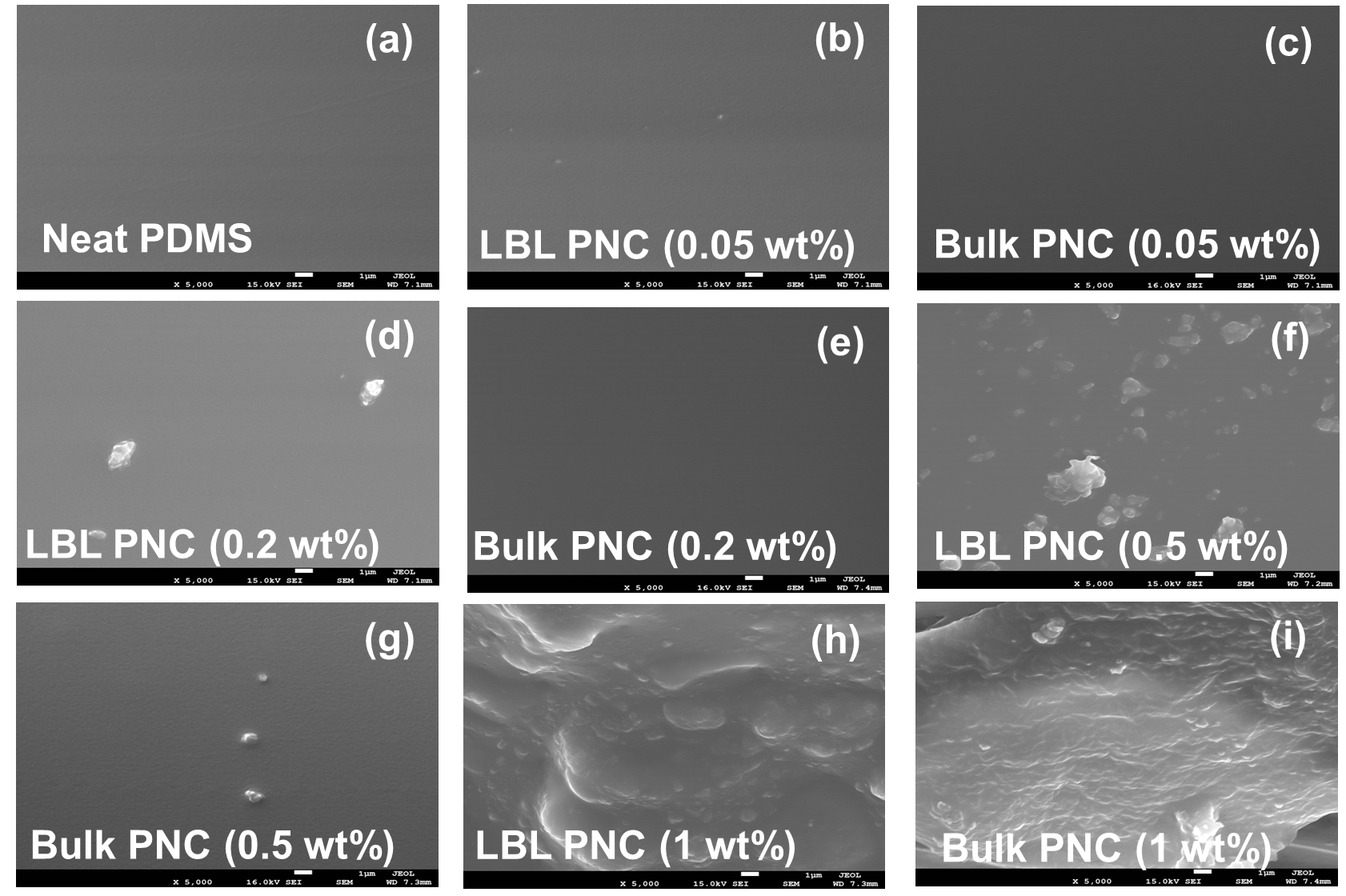
**

**Figure S1.** Top surface FESEM imaging of the LBL and bulk PNC samples: (a) Neat PDMS, (b) LBL PNC (0.05 wt.%), (c)Bulk PNC (0.05 wt.%), (d) LBL PNC (0.2 wt.%), (e) Bulk PNC (0.2 wt.%), (f) LBL PNC (0.5 wt.%), (g) Bulk PNC (0.5 wt.%), (h) LBL PNC (1 wt.%), and (i) Bulk PNC (1 wt.%).


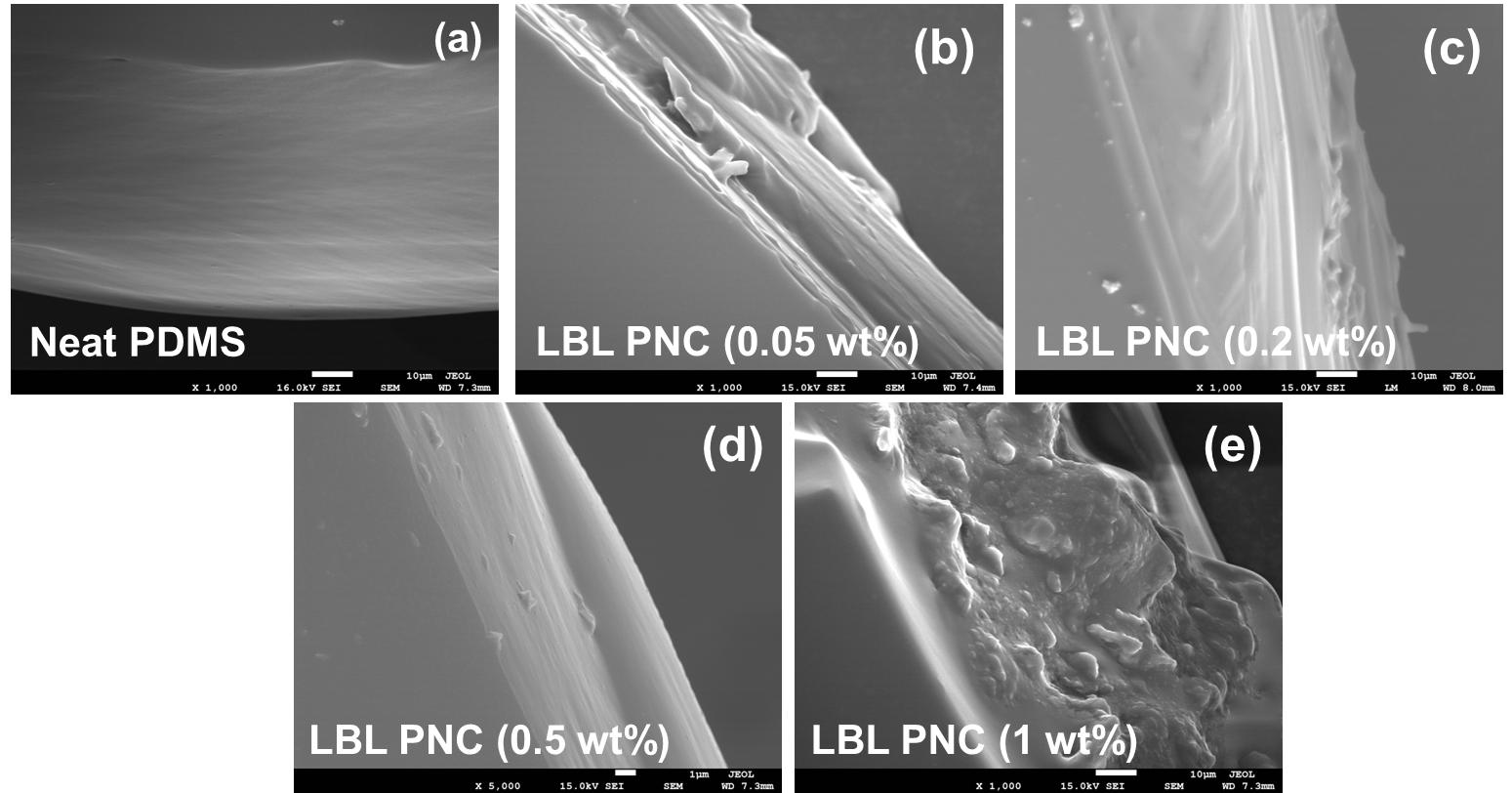


**Figure S2.** Cross-sectional image of the LBL PNC samples from a top-view: (a) Neat PDMS (b) LBL PNC (0.05 wt.%), (c) LBL PNC (0.2 wt.%), (d) LBL PNC (0.5 wt.%), (e) LBL PNC (1 wt.%).

The images in Fig. S2 are well in line with the cross-sectional analysis of the composite samples in Fig. 5 and 6 in the main text. As the CNT loading weight percentage increases, there is an increased number of protrusions of CNTs from the PDMS matrix that can be observed in the tilted cross-sectional view. The cross-sectional view of neat PDMS shows a homogeneous morphology. The cross-sectional view of the LBL PNC (1 wt.%) (Fig S2 (e)) loading shows a high number of protrusions of SWCNTs protruding outwards from the PDMS matrix. The LBL assembly of alternating layers of PDMS and SWCNT can also be clearly seen when compared with the neat PDMS film.

**S2. Nanoindentation Analysis and Mechanics of Viscoelastic Behavior**

**S.2.1 Correlation of elastic modulus (*E*) from reduced modulus (*E_r_*) values**

The reduced modulus (*E_r_*) of the sample is a parameter that is generated during quasi-static nanoindentation test. It is represented as:

$E_{r}= \frac{S\surd\pi}{2 \surd A_{c}}$ (S1)

where *S* is the slope of the upper portion of the unloading curve and *A_c_* is the tip contact area, which for the perfect Berkovich tip is related to the indentation depth, h, as:

$A_{c}=24.5 h^{2}$ (S2)

The tip area function is derived from indentations made into a fused quartz standard sample with a reduced modulus of 69.6 GPa. The obtained reduced modulus, *E_r_*, is correlated with the elastic modulus of PDMS, *E_PDMS_*, according to:

$\frac{1}{E_{r}}= \frac{(1- \vartheta_{PDMS}^{2})}{E_{PDMS}}+ \frac{(1- \vartheta_{tip}^{2})}{E_{tip}}$ (S3)

*υ_PDMS_* represents the Poisson's ratio of PDMS (0.5), while *υ_tip_* represents the Poisson's ratio of the diamond indenter (0.07). Due to the significant difference in elastic moduli between the diamond indenter tip (1140 GPa) and PDMS (in MPa), the second term in equation (S3) is negligible. The relationship between the PDMS elastic modulus and the reduced modulus is therefore:

$E_{PDMS}= E_{r}\left( 1- \vartheta_{PDMS}^{2} \right)=0.75 E_{r}$ (S4)

As calculated in Equation S4, the reduced modulus of PDMS revealed through nanoindentation analysis is 25% more than the actual elastic modulus of the material. This is particularly important to consider when comparing the reduced modulus values from nanoindentation results with other testing methods, such as macroscopic compression or tensile tests. ^1,2^

**S.2.2 Estimation of contact stiffness (*S_c_*) of thin film samples estimated through nanoindentation analysis**

The contact stiffness (*S_c_*) was determined by the regression function fitted to the upper part of the unloading curve. ^3^ The elastic modulus of the indented sample can be inferred from the initial unloading contact stiffness, *S= dP/dh*, i.e., the slope of the initial portion of the unloading curve. Based on relationships developed by Sneddon *et al*., ^4^ for the indentation of an elastic half-space by any punch that can be described as a solid revolution of a smooth function, a geometry-independent relation involving contact stiffness, contact area, and elastic modulus can be derived as follows:

$S_{c}= 2\beta A_{c}\pi E_{r}$ (S5)

Where *β* ^5,6^ is a constant that depends on the geometry of the indenter (*β*=1.034 for a Berkovich indenter)

**S.2.3 Interaction of Berkovich tip with PDMS-SWCNT composite samples**

The Berkovich tip is a specialized type of nanoindenter tip used to determine the indentation hardness of materials. Its shape resembles a three-sided pyramid with geometric properties that are self-similar. The Berkovich tip is well-known for its flat profile, with an included angle of 142.3° and a half angle of 65.27° measured from the axis to one of the pyramid flats. Interestingly, the Berkovich tip has the same projected area-to-depth ratio as a Vickers indenter. With its three-sided design, the Berkovich tip can be sharpened to a point easily, making it ideal for nanoindentation tests. Typically, this type of tip is utilized to measure the hardness of bulk materials and films that are over 100 nm thick. A schematic representation of the Berkovich tip used for nanoindentation tests can be found in Fig. S3.


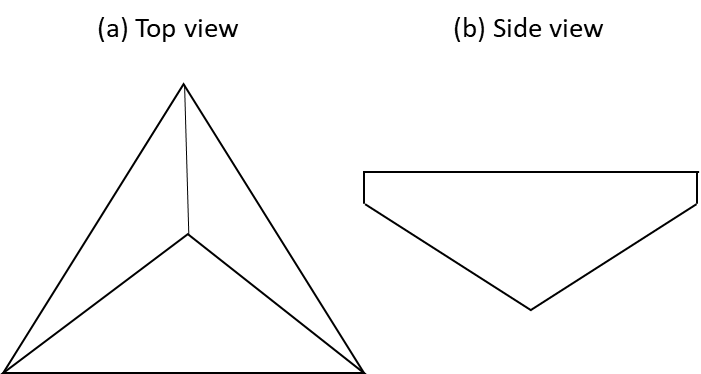


**Figure S3.** Schematic of Berkovich tip used for nanoindentation analysis of thin films.

A comparative schematic of the interaction of the Berkovich nanoindentation tip with the thin films is schematically represented in Fig. S4. As seen in Fig. S4, the LBL PNCs have well-distributed SWCNT on their top surface. In contrast, bulk PNC composites do not have well-distributed SWCNT on the top surface of the composites. In the case of the bulk PNCs, the tip encounters a lot of PDMS than SWCNT, as depicted by their inferior mechanical properties compared to LBL PNCs in nanoindentation results (Main text: Fig. 9 and Fig. 10) and the modulus, stiffness, and hardness results (Main Text: Fig. 11), which are discussed in detail in the main text.


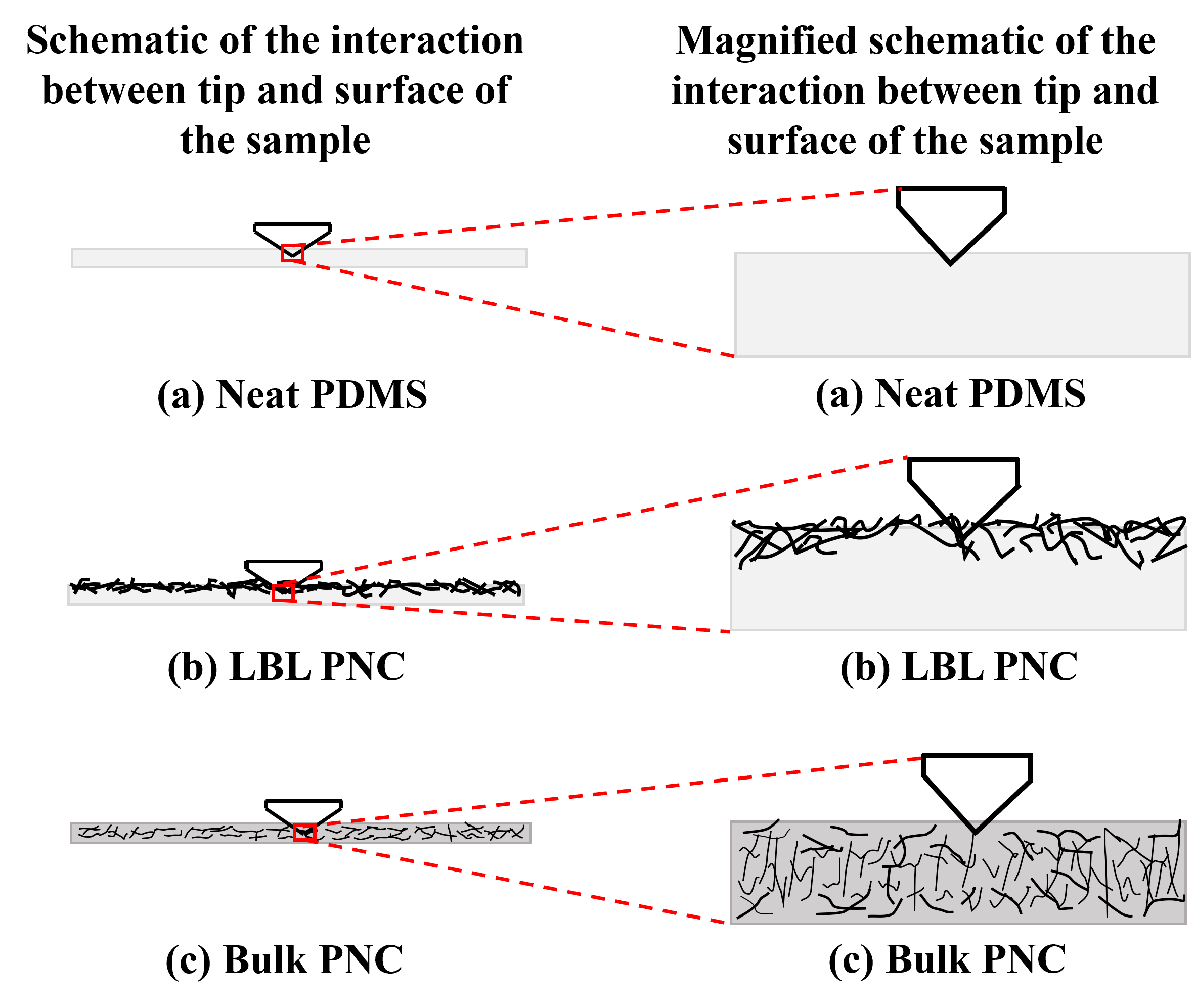


**Figure S4.** Schematic of the interaction between nanoindentation tip and the top surface of the thin film composites (a) Neat PDMS (b) LBL PNC (c) Bulk PNC.

**S.2.4 Force-Displacement Curves from Nanoindentation Analysis**

Figs. S5 (a)-(e) and Figs. S6 (a)-(d) shows 9 individual plots with 3 representative curves respectively from the 9 thin film samples: (i) Neat PDMS, (ii) Bulk PNC (0.05 wt.%), (iii) LBL PNC (0.05 wt.%), (iv) Bulk PNC (0.2 wt.%), (v) LBL PNC (0.2 wt.%), (vi) Bulk PNC (0.5 wt.%), (vii) LBL PNC (0.5 wt.%), (viii) Bulk PNC (1 wt.%), and (ix) LBL PNC (1 wt.%). The average of the area under each of these representative curves is calculated and is discussed in section 3.3.1 (Fig. 10) in the main text.

**S.2.5 Hardness and Reduced Modulus- Individual maps**

Figs. S7- S10 represent the reduced modulus (*E_r_*) and hardness (*H*) maps comprising the individual data points as obtained from each point of nanoindentation on the thin film samples. The box plots in Fig. 11 (a) and (c) in the main text is a summary of the data obtained from this raw information. As discussed sections S.2.1 and S.2.2, the elastic modulus (*E*) and contact stiffness (*S_c_*) are calculated from the reduced modulus (*E_r_*) and the slope of the upper portion of the unloading curve from each indentation point on the samples.


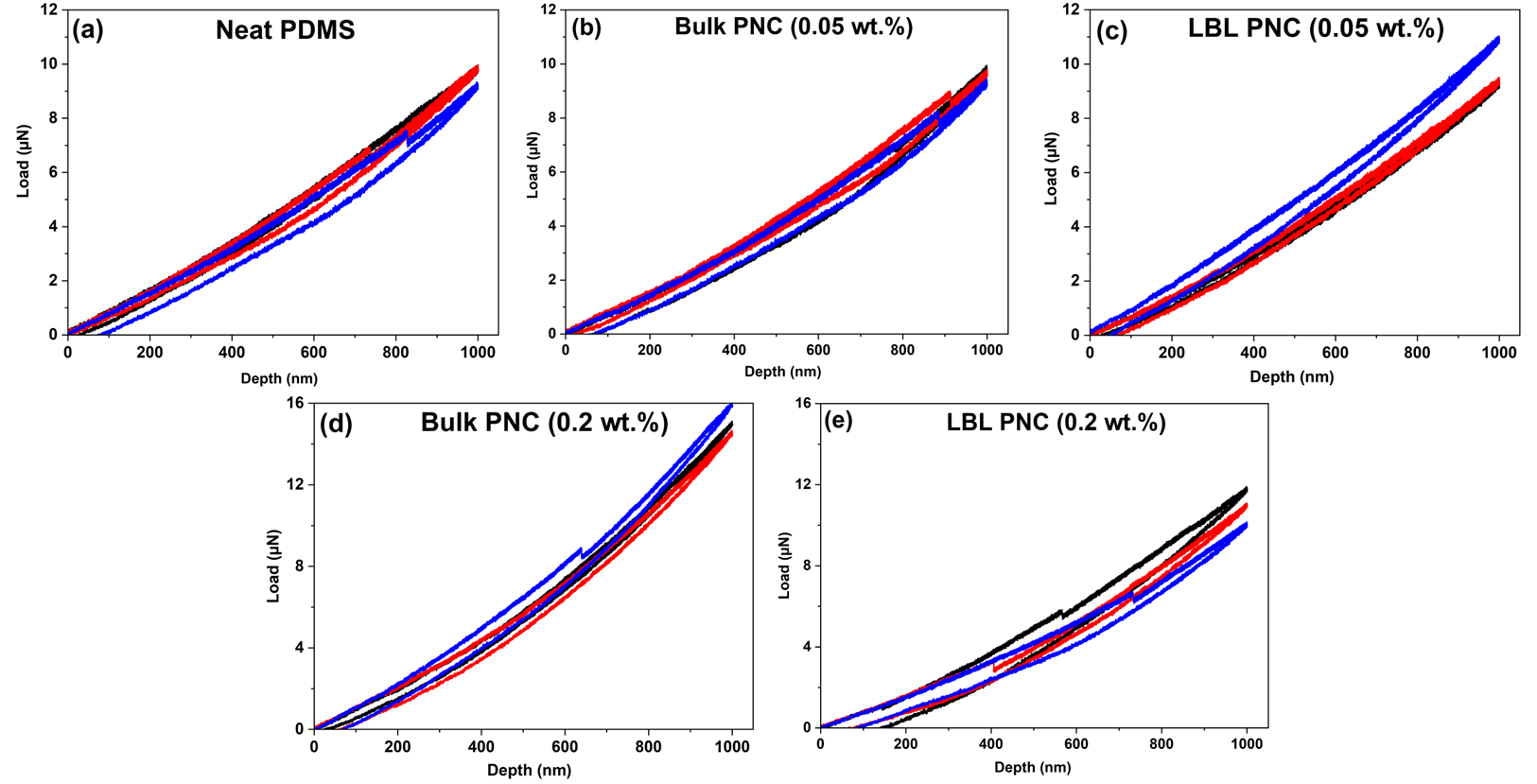


**Figure S5.** Loading- unloading behaviour of the thin film samples estimated through nanoindentation analysis: (a) Neat PDMS, (b) Bulk PNC (0.05 wt.%), (c) LBL PNC (0.05 wt.%), (d) Bulk PNC (0.2 wt.%), (e) LBL PNC (0.2 wt.%).





**Figure S6.** Loading- unloading behaviour of the thin film samples estimated through nanoindentation analysis: (a) Bulk PNC (0.5 wt.%), (b) LBL PNC (0.5 wt.%), (c) Bulk PNC (1 wt.%), and (d) LBL PNC (1 wt.%).

**
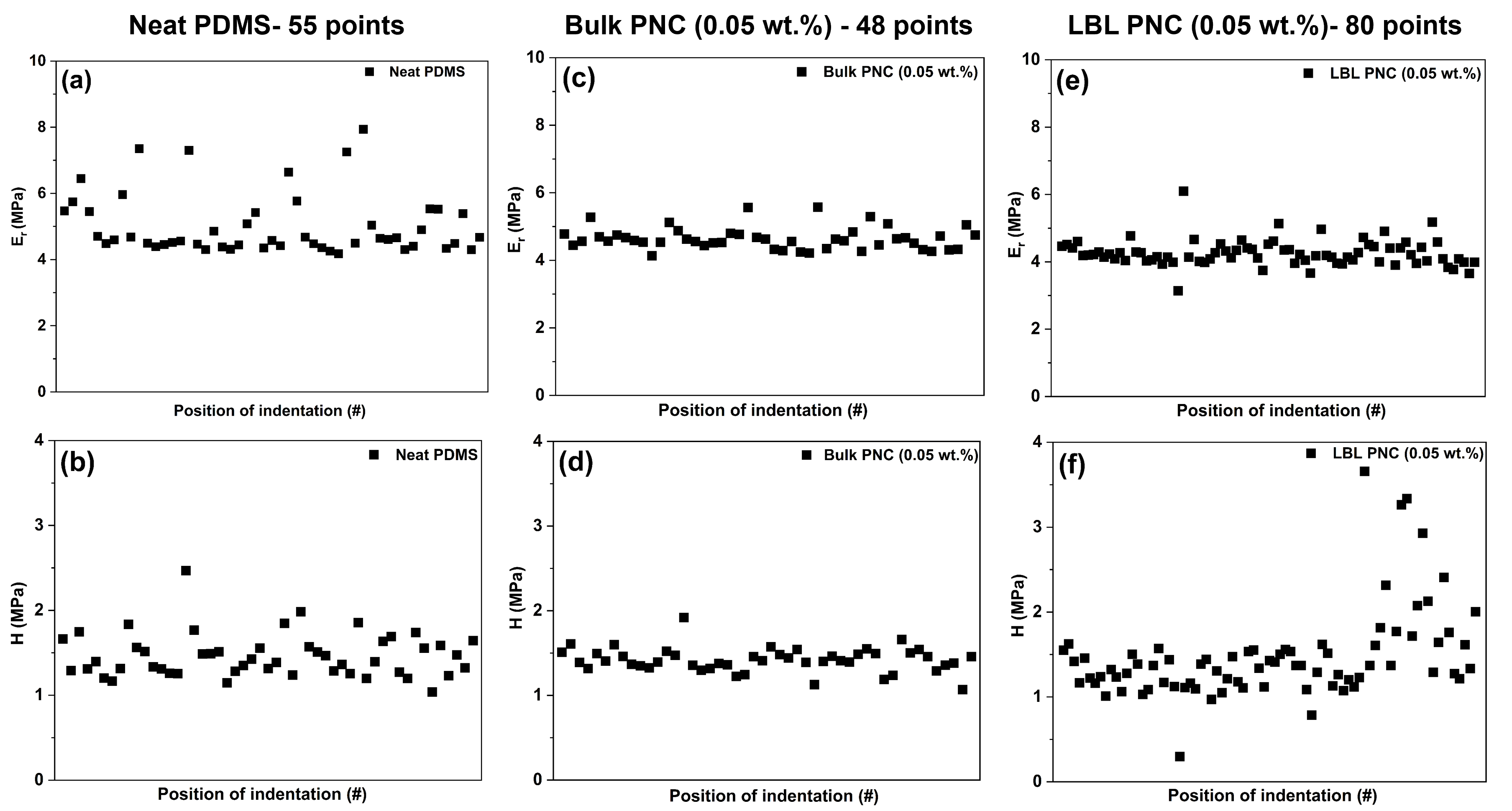
**

**Figure S7.** Individual reduced modulus and hardness maps of neat PDMS, Bulk PNC (0.05 wt.%) and LBL PNC (0.05 wt.%). The number of points tested for each sample is mentioned in the title of each plot.

**
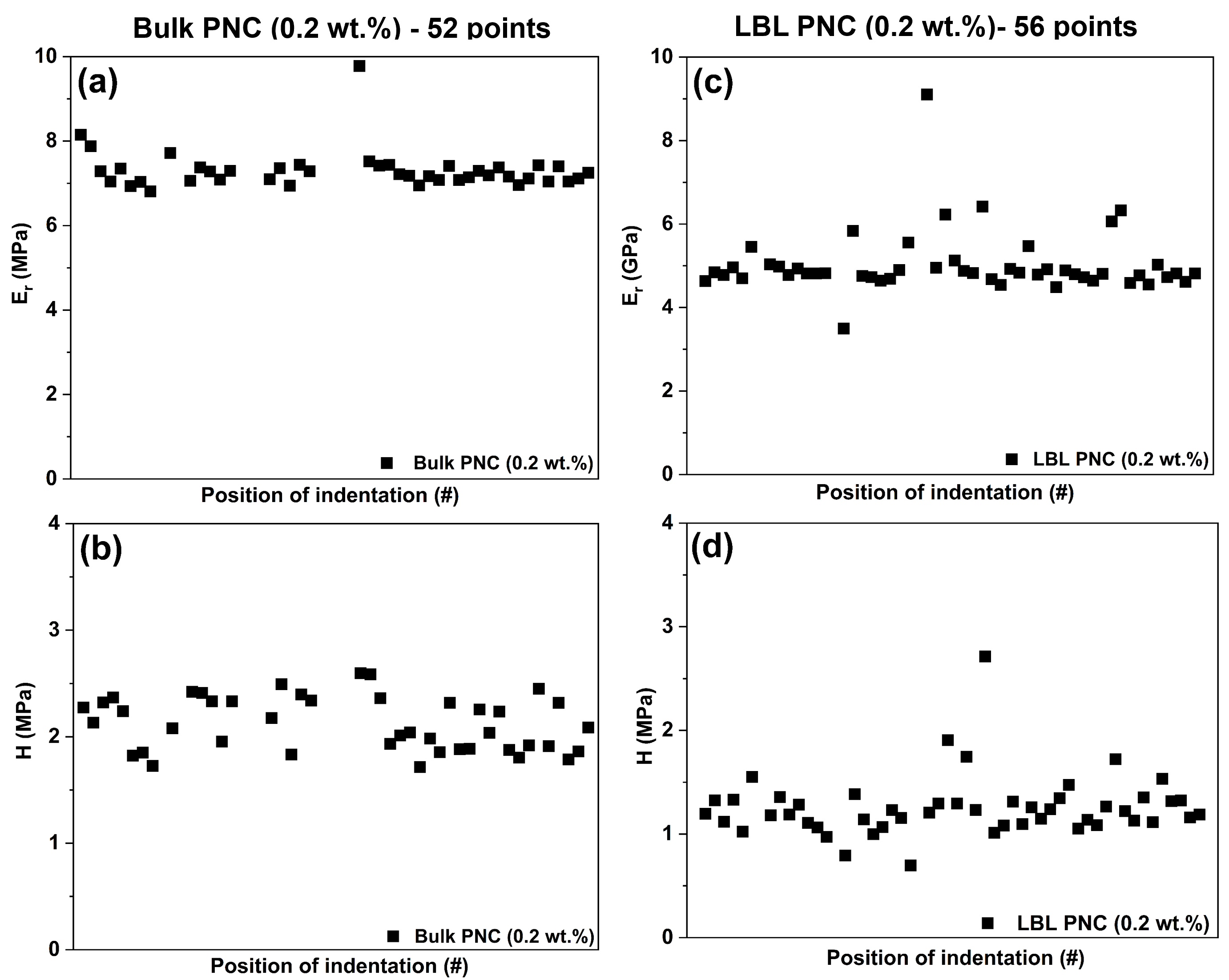
**

**Figure S8.** Individual reduced modulus and hardness maps of Bulk PNC (0.2 wt.%) and LBL PNC (0.2 wt.%). The number of points tested for each sample is mentioned in the title of each plot.

**
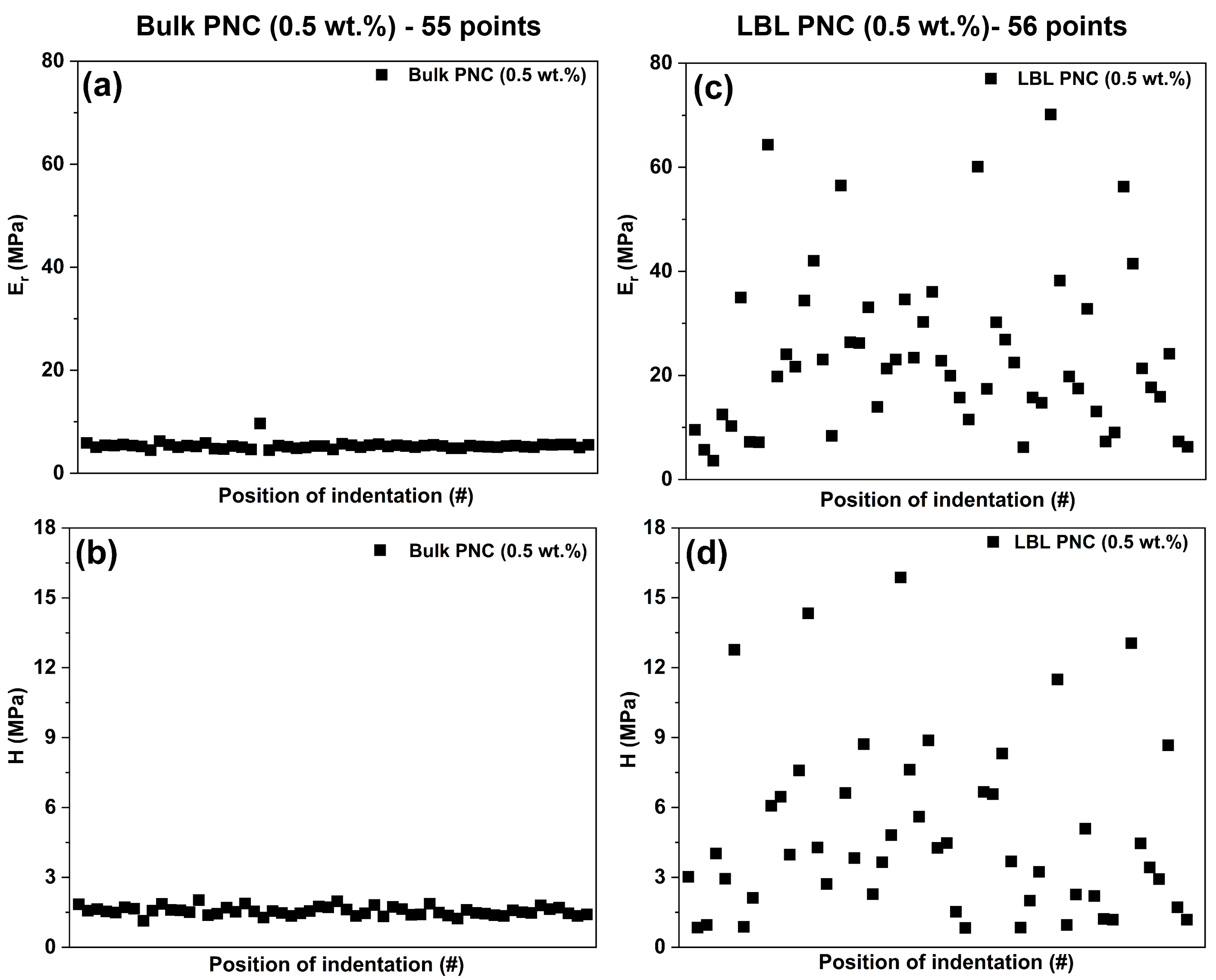
**

**Figure S9.** Individual reduced modulus and hardness maps of Bulk PNC (0.5 wt.%) and LBL PNC (0.5 wt.%). The number of points tested for each sample is mentioned in the title of each plot.

**
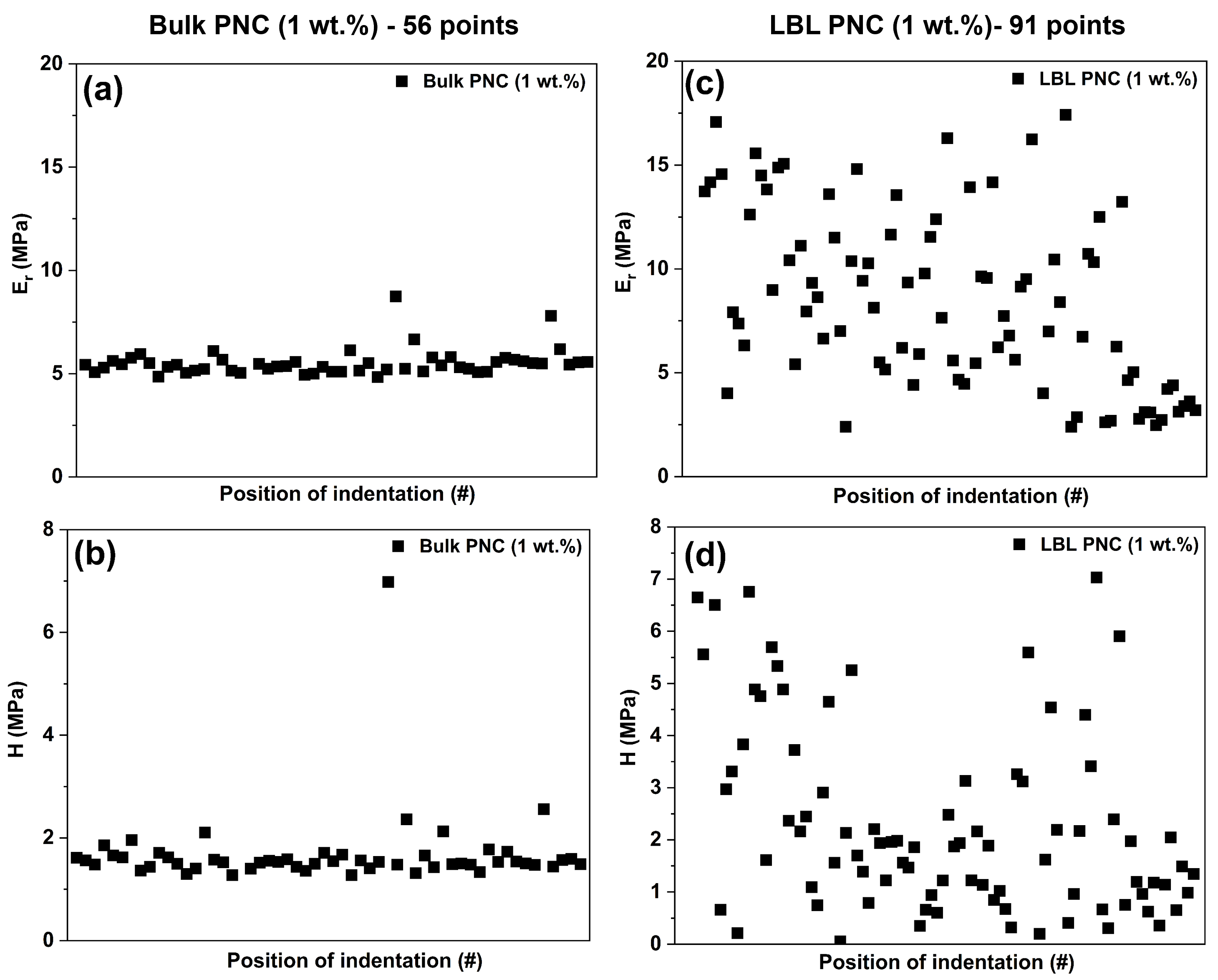
**

**Figure S10.** Individual reduced modulus and hardness maps of Bulk PNC (1 wt.%) and LBL PNC (1 wt.%). The number of points tested for each sample is mentioned in the title of each plot.

**REFERENCES**

1 Wang, Z., Volinsky, A. A. & Gallant, N. D. Nanoindentation study of polydimethylsiloxane elastic modulus using Berkovich and flat punch tips. *Journal of Applied Polymer Science* **132**, (2015).

2 Guillonneau, G., Kermouche, G., Bergheau, J.-M. & Loubet, J.-L. A new method to determine the true projected contact area using nanoindentation testing. *Comptes Rendus Mécanique* **343**, 410-418 (2015).

3 Menčík, J. in *Emerging Nanotechnologies in Dentistry* 307-326 (Elsevier, 2018).

4 Sneddon, I. N. The relation between load and penetration in the axisymmetric Boussinesq problem for a punch of arbitrary profile. *International journal of engineering science* **3**, 47-57 (1965).

5 Oliver, W. C. & Pharr, G. M. An improved technique for determining hardness and elastic modulus using load and displacement sensing indentation experiments. *Journal of materials research* **7**, 1564-1583 (1992).

6 Li, X. & Bhushan, B. A review of nanoindentation continuous stiffness measurement technique and its applications. *Materials characterization* **48**, 11-36 (2002).

**List of Figure Captions**

| Figure S1 | Top surface FESEM imaging of the LBL and bulk PNC samples: (a) Neat PDMS, (b) LBL PNC (0.05 wt.%), (c)Bulk PNC (0.05 wt.%), (d) LBL PNC (0.2 wt.%), (e) Bulk PNC (0.2 wt.%), (f) LBL PNC (0.5 wt.%), (g) Bulk PNC (0.5 wt.%), (h) LBL PNC (1 wt.%), and (i) Bulk PNC (1 wt.%). |
| --- | --- |
| Figure S2 | Cross-sectional image of the LBL PNC samples from a top-view: (a) Neat PDMS (b) LBL PNC (0.05 wt.%), (c) LBL PNC (0.2 wt.%), (d) LBL PNC (0.5 wt.%), (e) LBL PNC (1 wt.%). |
| Figure S3 | Schematic of Berkovich tip used for nanoindentation analysis of thin films. |
| Figure S4 | Schematic of the interaction between nanoindentation tip and the top surface of the thin film composites (a) Neat PDMS (b) LBL PNC (c) Bulk PNC. |
| Figure S5 | Loading- unloading behaviour of the thin film samples estimated through nanoindentation analysis: (a) Neat PDMS, (b) Bulk PNC (0.05 wt.%), (c) LBL PNC (0.05 wt.%), (d) Bulk PNC (0.2 wt.%), (e) LBL PNC (0.2 wt.%). |
| Figure S6 | Loading- unloading behaviour of the thin film samples estimated through nanoindentation analysis: (a) Bulk PNC (0.5 wt.%), (b) LBL PNC (0.5 wt.%), (c) Bulk PNC (1 wt.%), and (d) LBL PNC (1 wt.%). |
| Figure S7 | Individual reduced modulus and hardness maps of neat PDMS, Bulk PNC (0.05 wt.%) and LBL PNC (0.05 wt.%). The number of points tested for each sample is mentioned in the title of each plot. |
| Figure S8 | Individual reduced modulus and hardness maps of Bulk PNC (0.2 wt.%) and LBL PNC (0.2 wt.%). The number of points tested for each sample is mentioned in the title of each plot. |
| Figure S9 | Individual reduced modulus and hardness maps of Bulk PNC (0.5 wt.%) and LBL PNC (0.5 wt.%). The number of points tested for each sample is mentioned in the title of each plot. |
| Figure S10 | Individual reduced modulus and hardness maps of Bulk PNC (1 wt.%) and LBL PNC (1 wt.%). The number of points tested for each sample is mentioned in the title of each plot. |
